# An observational study of ballooning in large spiders: Nanoscale multifibers enable large spiders’ soaring flight

**DOI:** 10.1371/journal.pbio.2004405

**Published:** 2018-06-14

**Authors:** Moonsung Cho, Peter Neubauer, Christoph Fahrenson, Ingo Rechenberg

**Affiliations:** 1 Technische Universität Berlin, Institut für Bionik und Evolutionstechnik, Berlin, Germany; 2 Technische Universität Berlin, Institut für Biotechnologie, Berlin, Germany; 3 Technische Universität Berlin, Zentraleinrichtung Elektronenmikroskopie, Berlin, Germany; University of North Carolina at Chapel Hill, United States of America

## Abstract

The physical mechanism of aerial dispersal of spiders, “ballooning behavior,” is still unclear because of the lack of serious scientific observations and experiments. Therefore, as a first step in clarifying the phenomenon, we studied the ballooning behavior of relatively large spiders (heavier than 5 mg) in nature. Additional wind tunnel tests to identify ballooning silks were implemented in the laboratory. From our observation, it seems obvious that spiders actively evaluate the condition of the wind with their front leg (leg I) and wait for the preferable wind condition for their ballooning takeoff. In the wind tunnel tests, as-yet-unknown physical properties of ballooning fibers (length, thickness, and number of fibers) were identified. Large spiders, 16–20 mg *Xysticus* spp., spun 50–60 nanoscale fibers, with a diameter of 121–323 nm. The length of these threads was 3.22 ± 1.31 m (*N* = 22). These physical properties of ballooning fibers can explain the ballooning of large spiders with relatively light updrafts, 0.1–0.5 m s^−1^, which exist in a light breeze of 1.5–3.3 m s^−1^. Additionally, in line with previous research on turbulence in atmospheric boundary layers and from our wind measurements, it is hypothesized that spiders use the ascending air current for their aerial dispersal, the “ejection” regime, which is induced by hairpin vortices in the atmospheric boundary layer turbulence. This regime is highly correlated with lower wind speeds. This coincides well with the fact that spiders usually balloon when the wind speed is lower than 3 m s^−1^.

## Author summary

Aerial dispersal of spiders, which is known as “ballooning,” enables spiders’ wide range of dissemination—sometimes transoceanic. However, little is known about the ballooning mechanism of spiders, due to the difficulty of observing the ballooning silks and little awareness of spiders’ ballooning flight itself. From our observations in the field and in the laboratory using a wind tunnel, we have characterized the heretofore unknown physical properties of spiders’ ballooning silks. These quantitative values can explain large spiders’ ballooning behaviors. Crab spiders use tens of nanoscale fibers for their aerial dispersal. In the air current, these nanoscale fibers are governed by low Reynolds number fluid dynamics, which means that the viscous force of the air is much more dominant than the inertial force of the air. Using this nano/microscale fluid dynamics, spiders can become airborne with a light updraft. From our wind measurements, we suggest that spiders use the organized updraft current in the turbulent wind. This may explain why spiders show the ballooning behavior at a low wind speed, as the updraft frequently exists in the low-speed regime of the fluctuating wind flows. This work represents the most rigorous investigation of spider ballooning to date and will inspire future research on the subject.

## Introduction

Some spiders from different families, such as Linyphiidae (sheet-weaver spiders), Araneidae (orb-weaving spiders), Lycosidae (wolf spiders), and Thomisidae (crab spiders), can disperse aerially with the help of their silks, which is usually called ballooning behavior [1–6]. There are 2 representative takeoff methods in ballooning flight: “tiptoe” and “rafting” [7–10]. If spiders perceive appropriate weather conditions for ballooning, they climb up to the highest position of a blade of grass or a branch of a tree and raise their abdomen as if standing on their tiptoes, in order to position the abdomen at the highest level, before spinning the ballooning lines. They release a single or a number of silks in the wind current and wait until a sufficient updraft draws their body up in the air. This is known as a “tiptoe” takeoff [9,10] (see S1 and S2 Figs). Another takeoff method is called “rafting,” in which spiders release the ballooning lines from a hanging position, relying on their drag line [7,8,10] (see S3 Fig). In these ways, some spiders can travel passively hundreds of kilometers and can reach as high as 4.5 km above sea level [11,12]. For example, one of the first immigrant species on new-born volcanic islands are known to be spiders [13–15]. Aerial dispersal of spiders is an influential factor on agricultural economy and ecology because spiders are highly ranked predators in arthropods and impact on a prey’s population [16]. Due to the spider’s incredible aerial dispersal ability, the physical mechanism of a spider’s flight has been questioned for a long time, not only in public media but also in scientific research [16–23].

Ballooning dispersal is efficiently used by spiderlings (young spiders, just a few days after eclosion from their eggs) to avoid cannibalism at their birth sites, which are densely populated by hundreds of young spiders, and to reduce competition for resources [23,24]. Some adult female spiders balloon to find a place for a new colony [4,25,26], and others balloon to search for food and mates [4,27]. Most of the ballooning spiders were spiderlings and spiders under 3 mm in length and 0.2–2 mg in mass [1–5,28,29]. Nevertheless, there are only a few reports on the ballooning of large spiders (over 3 mm in length, over 5 mg in mass) [4,5,25,26].

Spiders balloon most frequently during late spring and autumn seasons [2,4,30]. The influences of microclimates on ballooning—such as temperature, humidity, and wind conditions—have been extensively studied: (i) Many studies agree on a positive correlation of temperature [1,3,31] or a rapid increase in temperature [31–34]; (ii) low humidity is favorable for spiders to balloon [1,32,34]; (iii) for small spiders, 0.2–2 mm in length, the favorable mean wind speed is limited to 3 m s^−1^ at a level of 2 m [30,31,35]. The local favorable wind speeds were 0.35–1.7 m s^−1^ in experiments and 0.55–0.75 m s^−1^ in nature [1,3]. These values, however, differ for spiders of different sizes (between 0.78–1.21 mm) [1,36]. Recently, Lee and colleagues showed that not only the mean wind speed at a level of 2 m but also the local wind speed can be limited by a wind speed of 3 m s^−1^ for spiderlings [37]. Instability of atmosphere was pointed out as an influential factor [30,31,35]. Suter and Reynolds suggested a possible relation of spiders’ ballooning behavior with atmospheric turbulent flow [16,20].

There have been a number of models that have tried to explain spiders’ high buoyant capability (aerial dispersal capability): a fluid-dynamic lollipop model [17], a flexible filament model in turbulence [16], and an electrostatic flight model [22]. Recently, Zhao and colleagues implemented the 2-dimensional numerical simulation using an immersed boundary method, which can simulate the ballooning dynamics in more detail [38]. The result shows that the atmospheric instability enables longer suspension of a ballooner in the air, which agrees with the result of Reynolds’s simulation and suggests that a spider may sense the vibration of vortex shedding on the spider silk through their silk [16,38], which is an interesting hypothesis.

In spite of the abovementioned models and studies, dynamics in spiders ballooning are still not well understood, because of a lack of serious scientific observation studies and specific experiments. Many of the ballooning spiders are very small, with weights of 0.2–2 mg, which are difficult to study [1,3,36,37]. Many described experiments were not focusing on the spider’s ballooning behavior itself but assumed that spiders use a certain length of the drag line [18,19,21]. The ballooning of large spiders is also a struggle because (i) the observed physical properties of ballooning silks and spider size (60–80 cm long and 3–4 silk threads, 85–150 mg body weight) of an adult of *Stegodyphus mimosarum* seemed to be unrealistic for ballooning [25], because the required vertical speed of wind was 9.2–21.6 m s^−1^, according to Henschel’s calculation [18,39]; (ii) Humphrey’s model cannot explain the ballooning flight of spiders with a weight of over 9 mg, because of the mechanical properties of a spider silk [17]. The following questions are still to be answered: (i) How many and how long are the silk fibers needed for ballooning, especially in the case of large spiders with weights over 5 mg? (ii) Which silk fibers and glands are used for ballooning? (iii) How do ballooning silks shape during the flight? (iv) Do spiders control the buoyant capability by changing the length of silks or their pose during the flight? (v) Why do spiders usually balloon at a low wind speed (below 3 m s^−1^)?

The aim of this paper is to offer behavioral clues and quantitative data in ballooning flight that may answer these questions. Therefore, we investigated the ballooning behavior of adult and subadult crab spiders (*Xysticus* spp., Thomisidae) that had a size of 3–6 mm and a weight of 6–25 mg. This observation of large spiders could provide a good basis for the physical characterization of ballooning. Additional experiments were performed in a wind tunnel for a precise documentation of ballooning silks and to analyze the details of ballooning behavior. Also, the aerodynamic environment on a flat grass field was measured to investigate the usable updraft for a ballooning flight.

## Materials and methods

### Ethics statement

The species used in the experiments (*Xysticus* genus) are not endangered or protected species. No specific permissions were required. All applicable international, national, and institutional guidelines for the care and use of animals were followed. No permission was required to collect insects from the collection site, the Lilienthal Park and the Teltow Canal. These sites are within the province of Steglitz-Zehlendorf.

### Animal care

The collected adult and subadult crab spiders (*Xysticus* genus) were raised separately in a plastic box (length × width × height: 13 × 13 × 7 cm), which has ventilation holes. Once a week, the spiders were fed a mealworm, *Tenebrio molitor*, and moisture was provided with a water spray.

### Field observations

Crab spiders *(Xysticus cristatus*, *X*. *audax*, etc.) were collected at Lilienthal Park and along the Teltow Canal in the Berlin area, Germany, and observed each autumn, especially during October, from 2014 to 2016. Lilienthal Park was selected for the observation of preballooning behavior because the ballooning phenomenon of adult and subadult crab spiders is frequently observed in this region.

#### Takeoff

On sunny and partly cloudy days in autumn, 14 crab spiders (8 females, 2 males, and 4 not identified; adult or subadults) were collected at Lilienthal Park. These spiders were released in the same place on a self-built artificial mushroom-like platform (a 5.5 cm diameter half sphere, 1.2 m above a ground surface, gypsum material). This platform was intended to stimulate tiptoe preballooning behavior. Because of its convex surface (half sphere), spiders can easily recognize that the top of the convex surface is the highest position that may promote tiptoe behavior. The white color of this platform allowed the visual clarity of the spider’s behavior. During the observation, the ballooning behaviors were recorded by digital camera. Additionally, titanium tetrachloride was used for the flow visualization of the wind. The local wind speed and temperature were not measured directly, but the values from the Dahlem weather station (4.5 km distance from the observation site, the anemometer is installed 36 m above the ground in the 20 m-high forest canopy) may provide approximate conditions.

#### Statistical analysis of preballooning behaviors

The 14 crab spiders were set a total of 27 times onto the mushroom-shaped platform in natural weather. The detailed preballooning behaviors in “takeoff” observation were analyzed statistically: (i) active sensing motion, (ii) tiptoe motion, (iii) tiptoe takeoff, (iv) drop down, and (v) rafting takeoff (see “Takeoff” in the Results section). If they did not tend to balloon or hid for about 5 min, they were recollected. Once a spider raised 1 or both front legs (leg I) and then put them back again on the platform, this was considered to be an active sensing motion and was counted as 1 behavior. Tiptoe motion, raising the abdomen and putting it down again, was also counted as 1 motion. The transitions between behaviors were also counted. These numbers were first expressed as percentage frequency by normalizing with the total number of transitions (see Eq [1], *f*_*i*,*j*_ is a normalized frequency of the transition from i-th to j-th behavior. *n*_*i*,*j*_ is the number of the transition from i-th to j-th. *m* is the number of categorized behaviors). The probability from one behavior to next behavior is expressed by normalizing with the total number of previous behaviors (see Eq [2], *p*_*i*,*j*_ is the probability of the transition from i-th to j-th behavior).

$$f_{i,j}=\frac{n_{i,j}}{\sum_{i=1}^{m}\sum_{j=1}^{m}n_{i,j}},\;\sum_{i=1}^{m}\sum_{j=1}^{m}f_{i,j}=1$$ (1)

$$p_{i,j}=\frac{n_{i,j}}{\sum_{j=1}^{m}n_{i,j}}\;,\;\sum_{j=1}^{m}p_{i,j}=1,\;\text{where}\;i=1,2,\ldots ,m$$ (2)

#### Gliding

Gliding of crab spiders was observed in the autumn along the Teltow Canal because of the ecological and topographical benefits for the observation of ballooners: (i) crab spiders frequently glide along the canal during the autumn; (ii) the angle of the sun’s rays in the morning was appropriate for the detection of the ballooning silks during their flight; (iii) the dense trees on the opposite side of the canal provide a dark background, which facilitates the observation of floating silks. The shape of the threads during flight could not be photographed nor recorded as a video because only limited parts of the ballooning threads were reflected by the sun’s rays. However, the movement and shape of the threads could be recognized with the naked eye, and these shapes were quickly sketched by hand.

### Identification of ballooning lines

#### Sampling of ballooning lines

**Fig 1:**
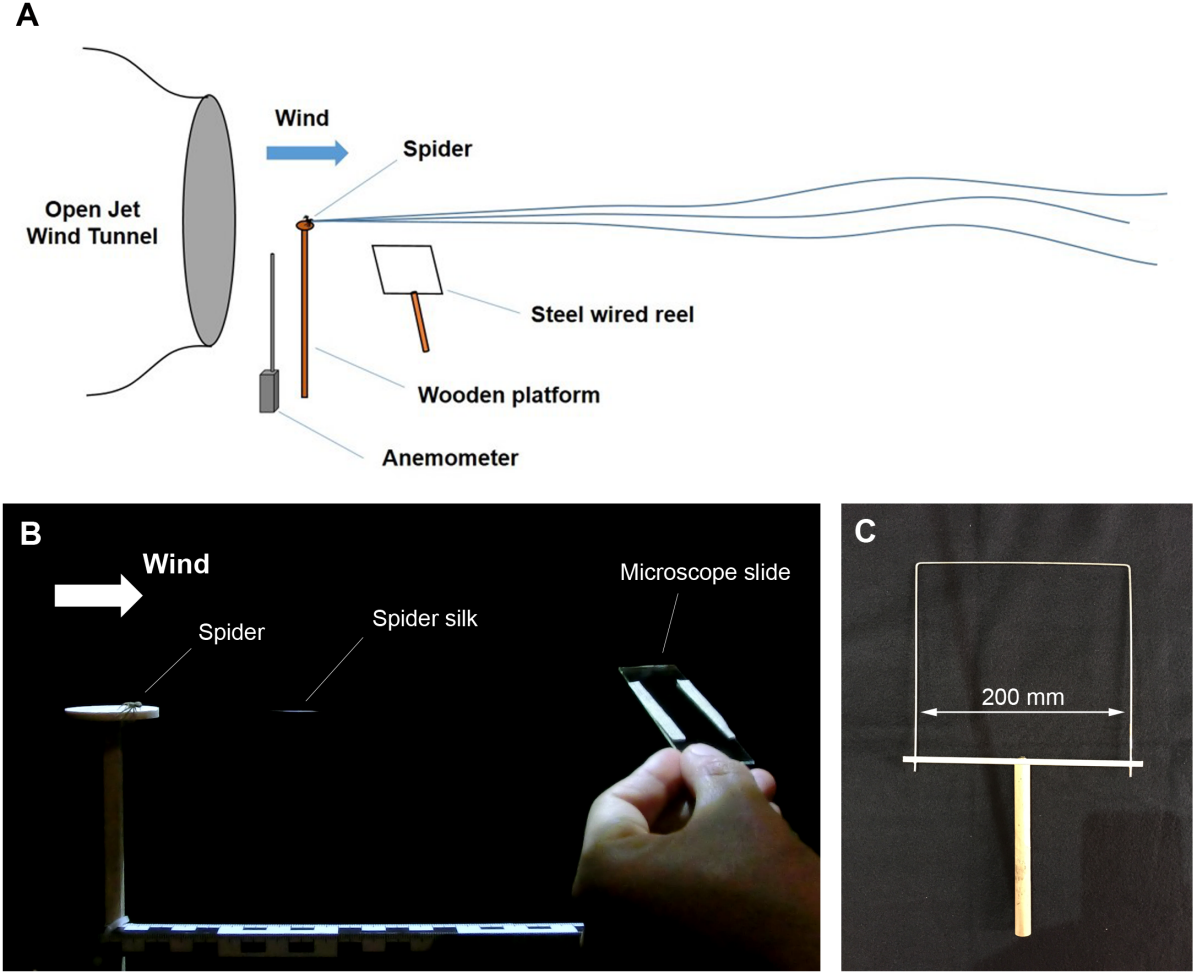
Experimental materials and methods for identification of ballooning lines. (A) A schematic view of wind tunnel tests. (B) Sampling of ballooning fibers in front of an open jet wind tunnel. (C) Reel with a steel wire to measure the length of ballooning silks.

Twelve crab spiders (9 females and 3 males, adult or subadult) were used for the wind tunnel experiment. Preballooning behavior of these spiders was induced in front of an open jet wind tunnel in which the diameter of the nozzle exit is 0.6 m (see Fig 1A). The spinning behaviors that led to the ballooning silks was observed precisely. There were no obstacles next to the wind tunnel, leaving about 9 m of free space from the nozzle to allow ballooning fibers to float horizontally without any adhesion to other objects. The wind speed and temperature were measured with a PL-135 HAN hot-wire anemometer. To enable sampling of the ballooning fibers, the wind speed was adjusted at 0.9 m s^−1^, because spiders drifted downstream if the wind speed was above 1 m s^−1^. The room temperature was 22–25 °C. The ballooning behavior was stimulated with a 1,000-watt hair dryer (low-wind-speed mode) that produces warm air (30–35 °C) and the fluctuation of wind. The hair dryer was positioned beneath the nozzle of the wind tunnel upward to avoid direct exposure to hot wind from the hair dryer (see S4 Fig). As soon as spiders showed tiptoe behavior, the hair dryer was turned off. The turbulent intensity of the wind tunnel was 1.1% (without the hair dryer) and 11.3% (when the hair dryer turned on). There was difference in temperature between the laboratory and the field. The difference can be explained as follows: first, the ballooning behavior is coupled not only with weather condition but also with biological condition, e.g., seasonal dispersal, mating, insufficient resources, etc. [2,4,26,30]. If the biological pressure for spiders to disperse is high, spiders may try to disperse even though it is low temperature. Second, the sudden increase of temperature acts on ballooning behavior as an influential factor [31–35]. If there is the sudden increase of temperature—e.g., because of sunshine in the morning—the ballooning behavior can be triggered even in low-temperature conditions. There are some reports that spiders ballooned also at relatively low temperatures, 10–20 °C [33,34; the author’s field observation (13–19 °C)].

Ballooning fibers were collected on a microscope slide on which 2 narrow strips of a double-sided bonding tape were attached. The ballooning fibers were sampled near the spinnerets (see Fig 1B). A total of 11 samples were prepared from 28 spinning events of ballooning silks. In the experiment, a total of 4 spiders responded to show ballooning behavior—two of them were very active. Two samples were selected, because the other samples failed to capture all the ballooning fibers on a single microscope slide or because the fibers on the slides were deranged during the capturing process. Simultaneously, silk fibers were captured on a square wire frame and carefully wound around it in order to measure the length of ballooning threads (see Fig 1C). The length of the silks was calculated by multiplying the total number of half revolutions by the width of the square wire frame (20 cm). The successfully sampled ballooning fibers were later studied with a field emission scanning electron microscope (FESEM).

#### FESEM

The sampled ballooning fibers were coated with gold using a sputter coater (SCD 030, Balzers Union) and observed with a FESEM (DSM 982 Gemini, ZEISS, with 5–10 kV accelerating voltage). The number of ballooning fibers was carefully counted, and the thickness of fibers was measured. The spinnerets of a female *X*. *cristatus* were also observed with the FESEM. For sample preparation, the female spider was fixed in 2.5% glutaraldehyde and dehydrated in ascending concentrations of ethyl alcohol from 30% to 100% (10 min at each concentration). After dehydration, the sample was dried with a critical point dryer (CPD 030, BAL-TEC). The prepared sample was coated with gold using a sputter coater (SCD 030, Balzers Union) and observed with the FESEM (SU8030, Hitachi, with 20 kV accelerating voltage).

### Investigation of the aerodynamic environment

**Fig 2:**
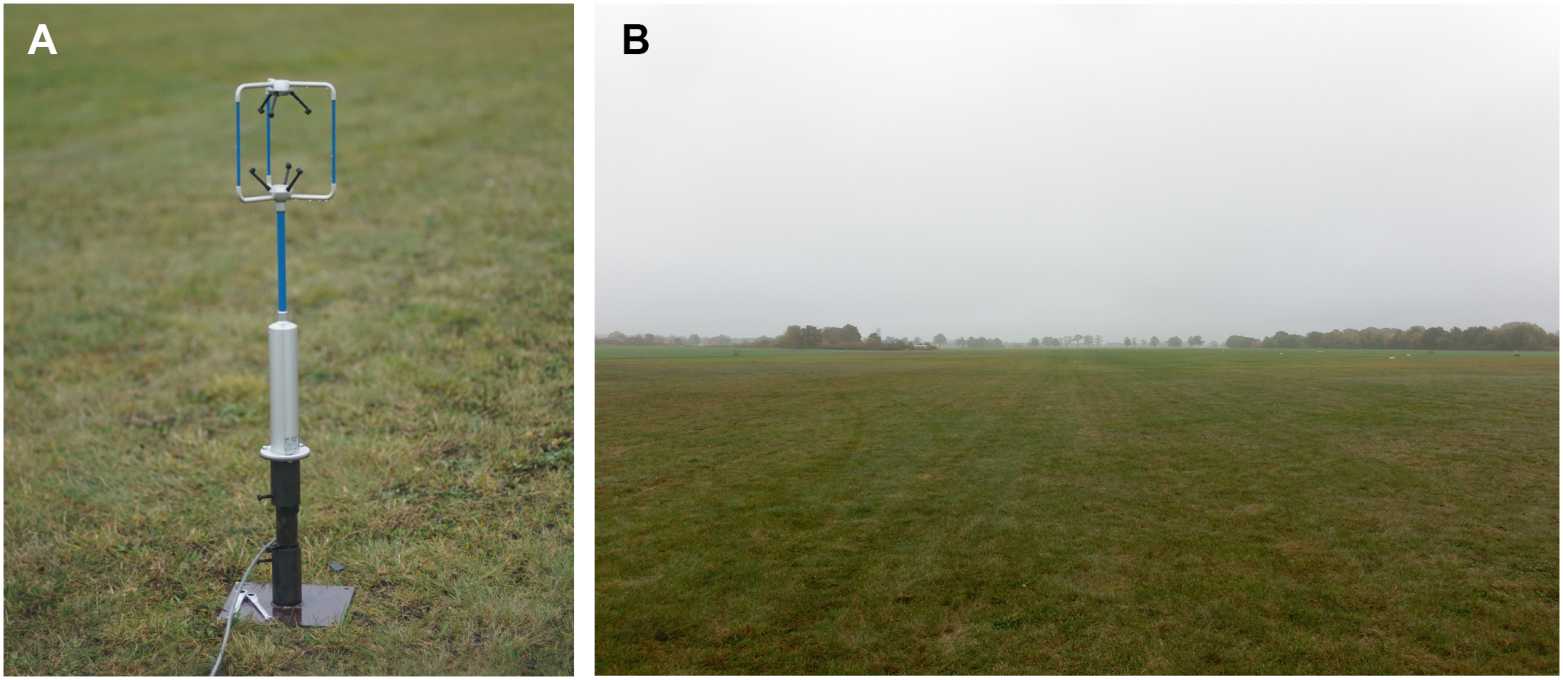
Experimental material and place for 3-dimensional wind velocity measurement. (A) A 3-dimensional ultrasonic anemometer (Windmaster 1590-PK-020, Gill Instruments) is installed 0.95 m above the ground. (B) The simplest conditions (i.e., a flat surface) were selected. The flat place is covered with the 6 cm short cut grass. Within a radius of 300 m, there is no obstacle object.

For the investigation of the turbulent atmospheric boundary layer, ultrasonic 3-dimensional wind speed measurement took place on a grass field (53° 11′ 42″ N, 12° 09′ 40″ E, see Fig 2A and 2B). This place is also a habitat of the *Erigone* and *Xysticus* genus, which do ballooning behavior. To avoid mechanically induced updrafts by hills, trees, and rocks, a flat grass field was selected. The 3-dimensional wind speed data at 2 different mean wind speeds (1.99 m s^−1^ and 3.07 m s^−1^) were measured on a sunny day in the autumn for 5 min with the sampling frequency of 20 Hz. In order to eliminate small-scale fluctuation in the turbulent boundary layer, a quadrant analysis was introduced [40–44] (see Eq [3]; *u* and *w* are, respectively, horizontal and vertical wind speeds. *u*′ and *w*′ represent, respectively, streamwise fluctuating velocity and vertical fluctuating velocity. Those are equal to the subtracted values of the actual values of velocity minus the mean values of the velocity. *H* is the threshold parameter, which means the size of a hole. $u_{rms}^{\prime }$ and $w_{rms}^{\prime }$ indicate root mean squared fluctuation velocities).

$$u^{\prime }w^{\prime }\geq \;H(u_{rms}^{\prime }w_{rms}^{\prime })$$ (3)

## Results

### Field observations

#### Takeoff

In the Thomisidae family, not only female but also male adult spiders showed ballooning behaviors (see S1 Fig). During the observation days, the temperature was 16–19 °C, and the mean wind speed was 6–7 m s^−1^ (gust 14–17 m s^−1^), as reported by the nearest weather station in Dahlem. The sensor was installed on 36 m position above the ground. Therefore, the local wind speed at 1.2 m above the ground must be much lower than these values. Later, we checked the wind speed on a similar day, on which the mean wind speed from the weather station showed 6–7 m s^−1^. The mean wind speed for 10 min showed 2.11 m s^−1^.

**Fig 3:**
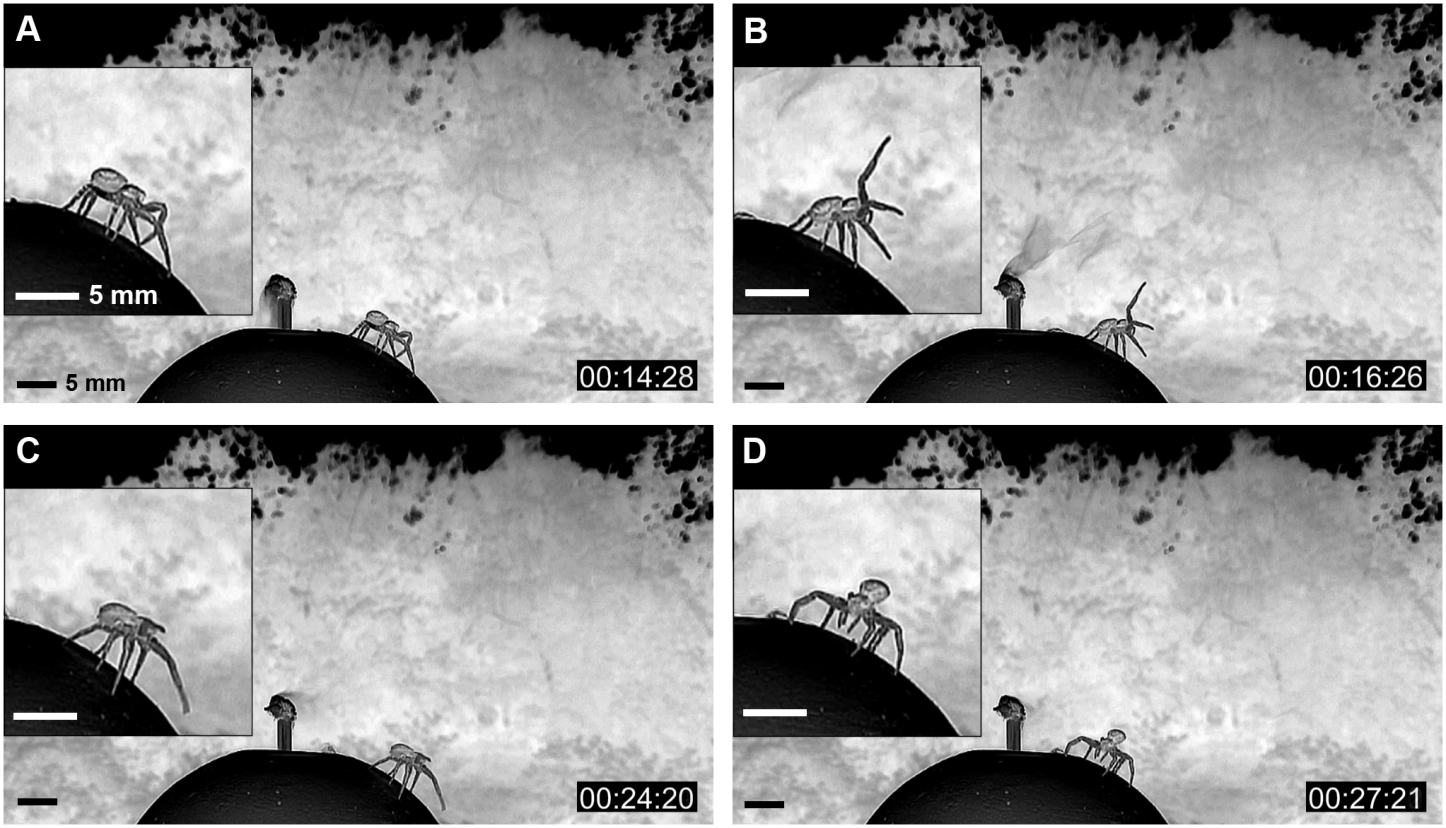
Sequence of active sensing motion with front leg (leg I) (negative images). (A) The spider first senses the condition of the wind current only through sensory hairs on its legs. (B) Then, if the condition seemed appropriate, the spider sensed more actively by raising leg I and keeping this pose for 8 sec. (C) If the spider decided to balloon, it altered its posture. (D) The spider rotated its body in the direction of the wind and assumed tiptoe posture.

On the experiment day, the spiders mostly showed tiptoe behavior. At the first stage of tiptoe behavior, the crab spider evaluated the wind condition not just passively through the sensory hairs on its legs but rather actively by raising 1 of its front legs (leg I), or sometimes both, and waited in this position for 5–8 s (see Fig 3A, 3B and 3C and S1 Video). This sensing behavior was often repeated a few times before the tiptoe pose. After each sensing step, the crab spider rotated its body in the direction of the wind (see Fig 3C and 3D).

If the spider decided that the wind was adequate to balloon, it raised its abdomen (already known as a tiptoe behavior, see Fig 3D) and spun its ballooning silks without any help from its legs. Before spinning ballooning silks, there was a motion of a rear leg (leg IV) (see S5 Fig), with which the spider holds its safety line that connected its spinnerets to the substrate and then puts it on the substrate (see S2A and S2B Fig and S2 Video).

**Fig 4:**
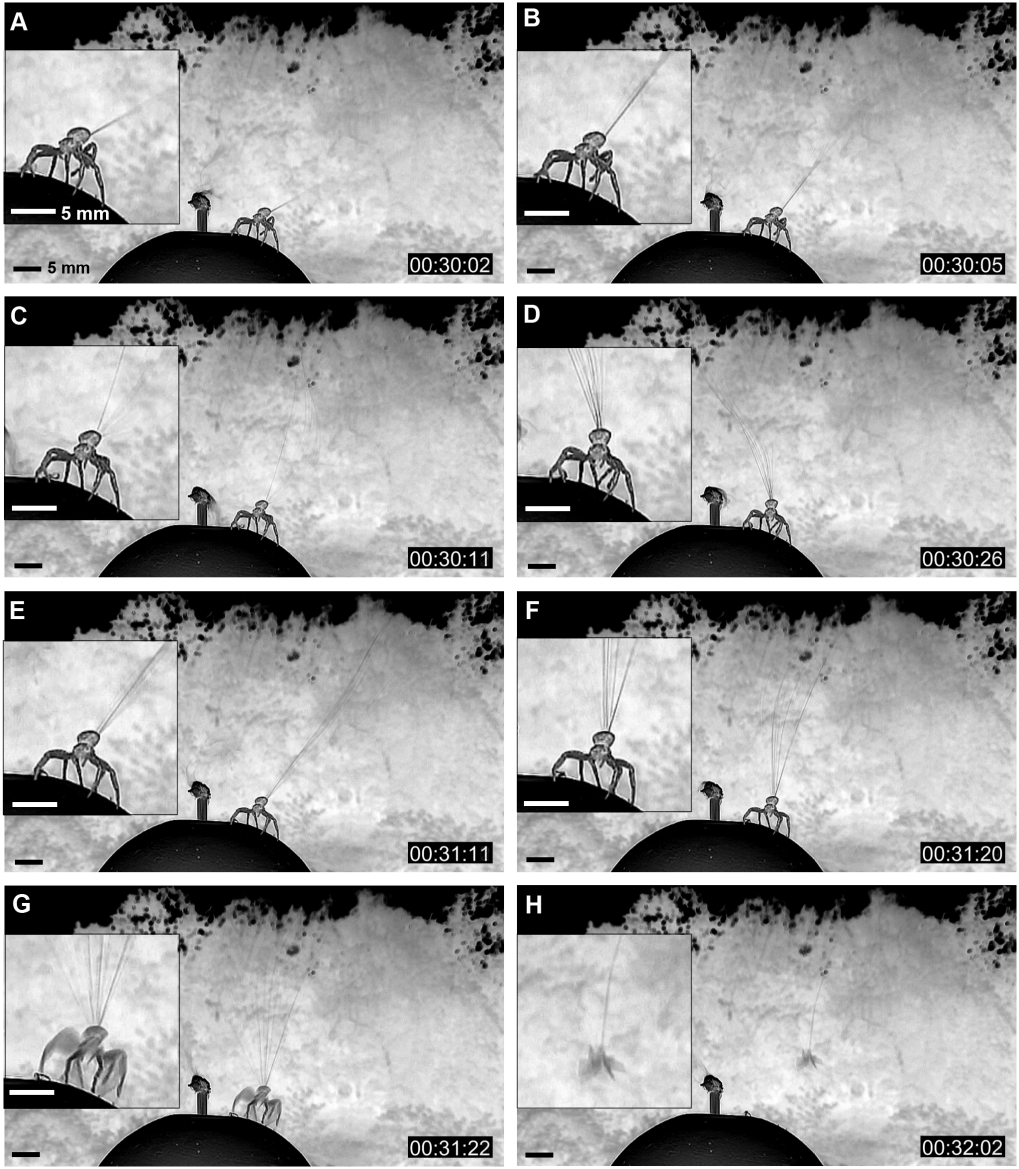
A crab spider’s ballooning process (images were converted to negative images to visualize ballooning lines). (A, B) Initial phase of spinning ballooning lines; (C, D, E, F) Fluttering of a bundle of ballooning lines. Because of turbulent flows in wind, the ballooning threads fluttered unsteadily. (G) Takeoff moment. (H) Airborne state of a ballooning spider. (Original video: see S3 Video).

The crab spider first spun a single or a few fibers and then many fibers (see Fig 4A–4G). The spun ballooning fibers were approximately 2–4 m long and formed a triangular sheet, which fluttered among the turbulent flows of the wind. The vertex angle of this triangular sheet was about 5–35° (see Fig 4C–4G). If the wind condition was not appropriate, the spider cut the silk fibers and spun them again. If the ballooning silks generated enough drag, the spider released the substrate and became airborne (see Fig 4H). From careful video investigation, it was observed that spiders stretched all their legs outward as soon as the spiders achieved takeoff. Many ballooned crab spiders soared diagonally upward along the wind flows. These paths had 5–20° inclination above the horizon. Some spiders traveled quasi-horizontally. Some spiders soared along a steep path (about 45°). During this steep takeoff, the spiders took off with relatively slow speed. The anchored drag line (safety line) between the platform and the spider’s spinneret could be seen. This anchored line endured without breaking until it became 3–5 m long. After a while, it was broken mechanically. From the wind tunnel experiment, it was found that the anchored line consists of 2 fibers.

Three new facts about ballooning were uncovered. First, the crab spider does not evaluate the wind condition passively, but actively by raising 1 of its legs I. Second, this adult ballooner anchors its drag lines on the platform not only during its rafting takeoff but also during tiptoe takeoff. Third, the crab spider postures all its legs outward and stretched, when airborne, not only at the takeoff moment but also during the gliding phase (see S6A–S6D Fig).

Rafting preballooning behavior was also observed. The local weather condition was a little bit colder and windier than that of the previous observation day of tiptoe takeoff. Crab spiders were not active on that day. As soon as they were set on the platform, they showed 1 of 2 behaviors. Either they hid on the opposite side of the platform to avoid the wind, or they quickly retreated downward about 0.4–1.1 m, relying on their drag lines (see S3A and S3B Fig), and spun their ballooning fibers downstream of the wind. At this time, spiders spun a single or a few fibers first and then many fibers, as they showed during the tiptoe takeoff (see S3C and S3D Fig). During this process, the spiders also postured all their front legs and second legs outward and backward so that they hung and directed their bellies in an upward direction of the wind. The backward (downstream)-spun threads slowly curved upward, and the spiders’ bodies also slowly moved upward (see S3D Fig). At some point, when the threads had generated enough drag and lift, the drag lines near the spinnerets were cut, and the spider finally ballooned (see S3E and S3F Fig).

#### Statistical analysis of preballooning behaviors

The spiders showed 68 active sensing motions with leg I. Forty-two tiptoe preballooning motions could be observed. Six spiders had ballooned successfully after their tiptoe pose. They also dropped down 8 times, relying on their drag line. Three of them showed a rafting takeoff (see Table 1).

**Table 1:** Ballooning behaviors on the artificial platform.

Date (2015)	Temp. (°C)	Wind (mean /gust) (m s^−1^)	Number of specimens	Number of tests	Sensing motion	Tiptoeing		Rafting		Total ballooned spiders	Escape (fall down)
						Tried	Ballooned	Drop & climb	Ballooned		
**9.24** (13:00–14:30)	19	6/17	7	14	48	30	4	1	0	4	3
**9.26** (13:00–15:30)	16 ± 1	7/14	5	11	20	12	2	5	1	3	2
**9.28** (12:00–12:30)	16	6/16	2	2	0	0	0	2	2	2	0
**Total**			14	27	68	42	6	8	3	9	5

**Fig 5:**
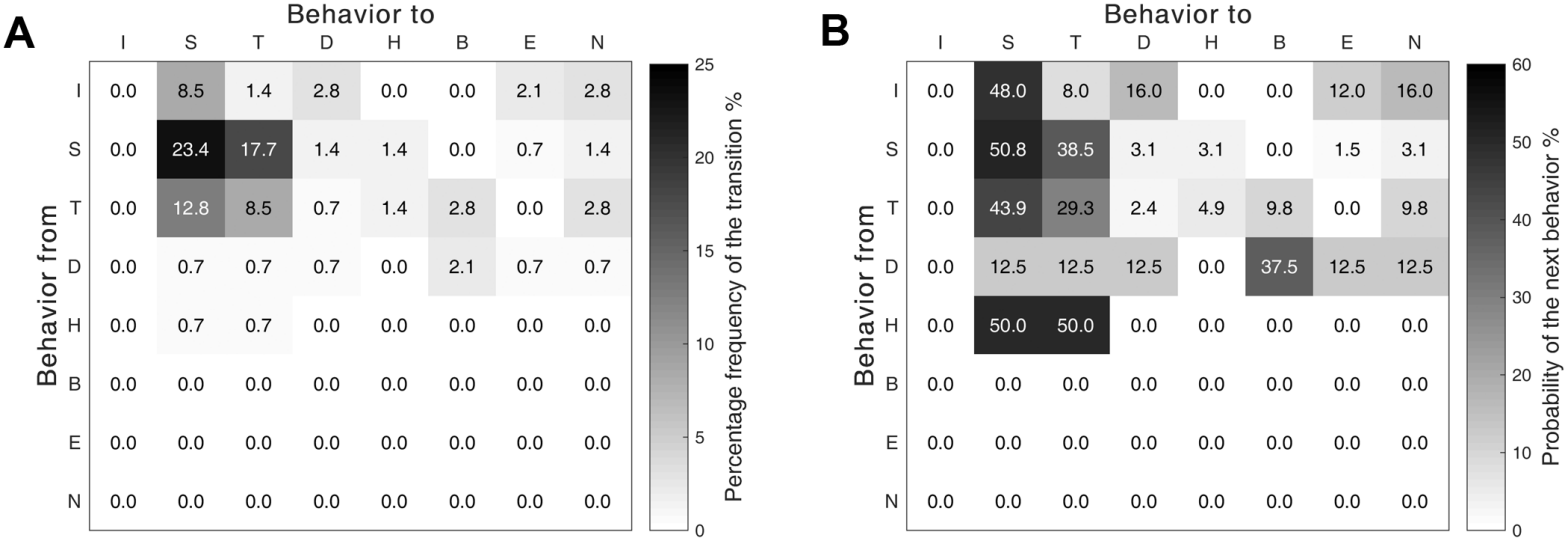
Sequential relations between behaviors for ballooning. (A) The percentage frequency of the behavior transition (the total number of transitions: *N* = 141). (B) The transition matrix between behaviors (the total numbers of categorized behaviors: *N*_*I*_ = 25, *N*_*S*_ = 65, *N*_*T*_ = 41, *N*_*D*_ = 8, *N*_*H*_ = 2). The corresponding underlying data can be found in S1 Data. B, takeoff; D, dropping and hanging behavior; E, escape; H, hiding motion; I, initial state; N, not flown; S, sensing motion; T, tiptoe behavior.

The initial behaviors were mostly started with sensing motion. The frequent transitions occurred between sensing motion and tiptoe motion (see Fig 5A). Spiders flew from only either the tiptoe pose or the dropping and hanging pose (see Fig 5B). The probability of the ballooning takeoff from tiptoe behavior was 9.8%. The probability of the ballooning takeoff from the rafting pose was 37.5%.

**Fig 6:**
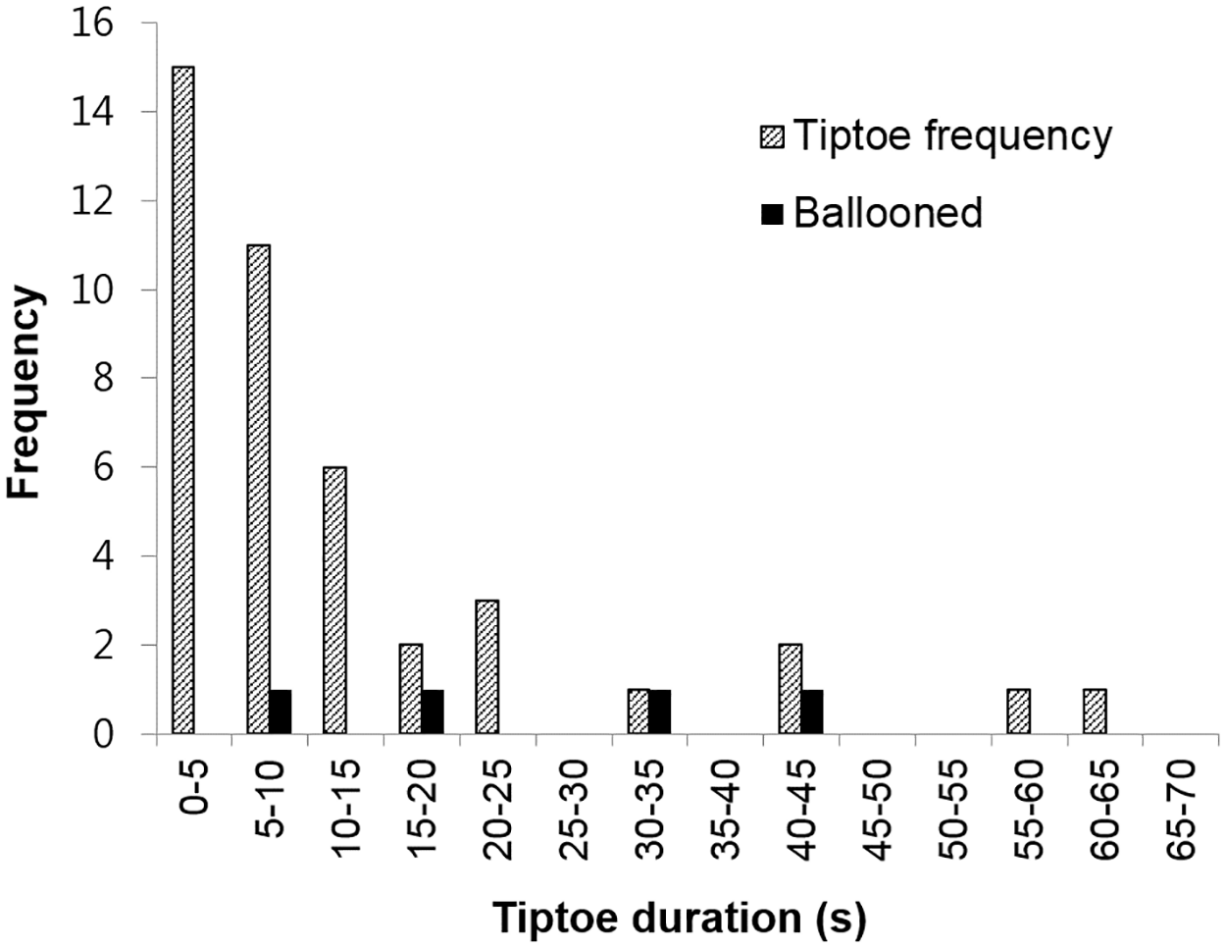
Frequency diagram of tiptoe behaviors according to tiptoe duration (*N* = 42). Black columns are the tiptoe behaviors that were connected to the successful ballooning takeoffs (*N* = 4). The corresponding underlying data can be found in S1 Data.

The duration of each tiptoe behavior was measured, and their frequencies were analyzed. Short-period tiptoe poses, which lasted for less than 5 s, were the most frequent. The longest tiptoe event lasted 65 s. The observed successful ballooning takeoffs were not biased in relation to tiptoe duration (see Fig 6).

#### Gliding

**Fig 7:**
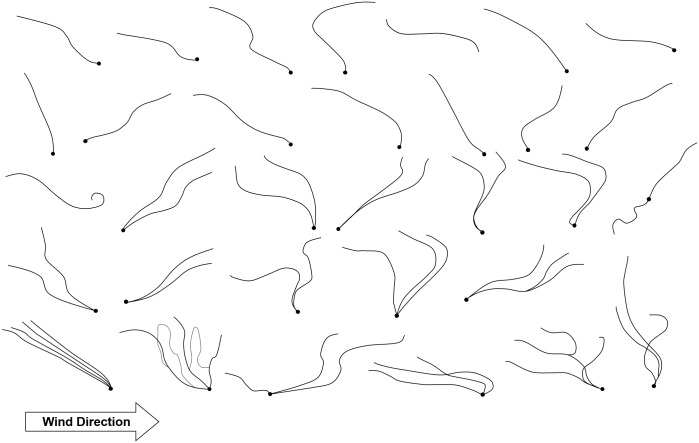
Sketches of ballooning structures (body + ballooning threads). These structures were observed above the water surface, at heights of 1–8 m. Wind was blowing from left to right. Therefore, these structures were transported in the same direction as the wind. Black, thick points represent the spider’s body. Black lines represent ballooning threads.

A total of 32 floating threads were observed at the Teltow Canal. Most of them were horizontally transported along the channel at about 1–8 m above the water surface. They drifted passively due to light wind but rarely fell down. Some of their silks were inclined downstream. The others were inclined upstream. Two of 32 floating threads were just threads alone without a spider. The number of observed threads was 1–5. However, as not all threads were visible with the naked eye, some may have been the multiple threads that stuck together, although they seemed to be a single thread. Some of them may not have been seen because of their inappropriate angles and positions in relation to the sun. Most of the spiders were positioned at the lower end of their threads. Although the threads showed different numbers and shapes, they were usually laid diagonally (see Fig 7).

### Identification of ballooning fibers

**Fig 8:**
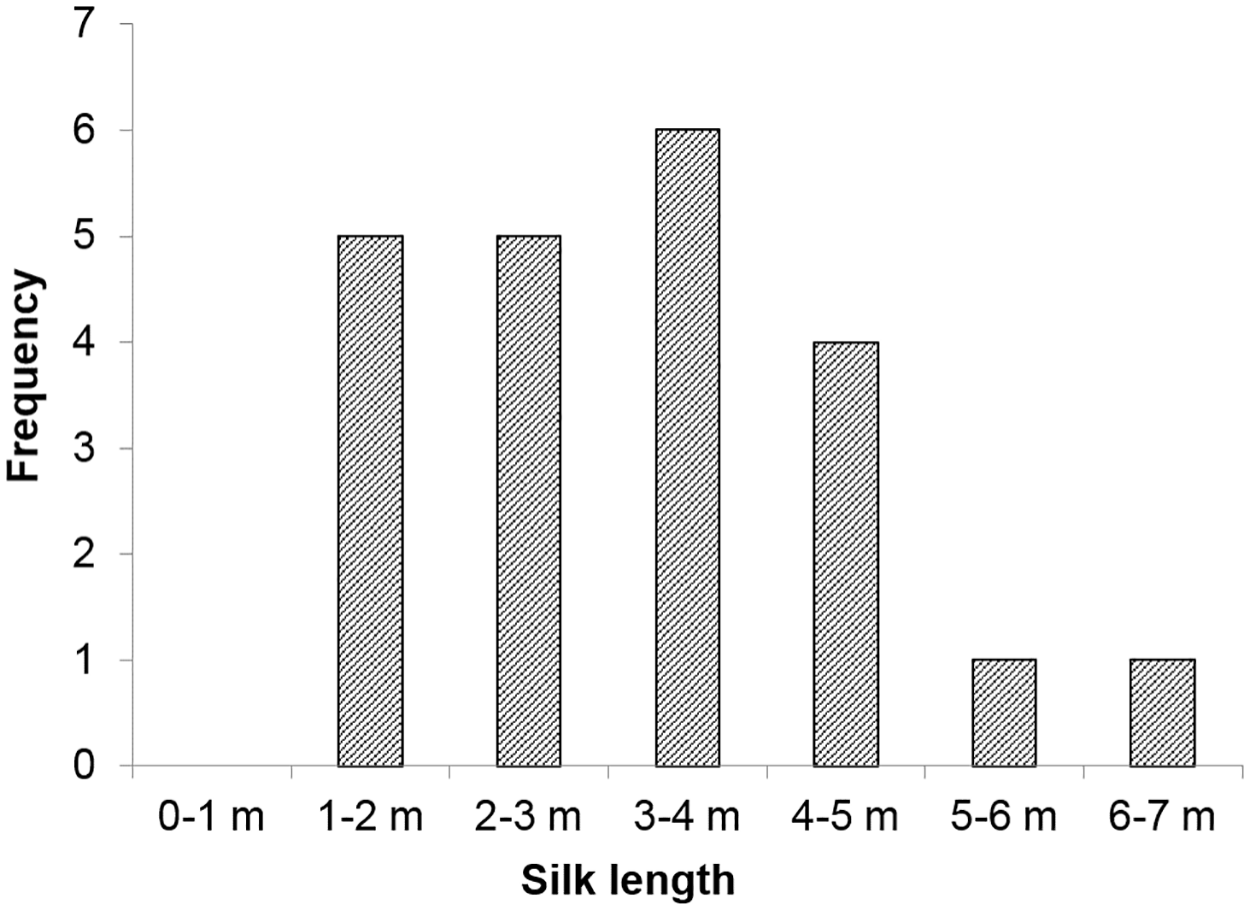
Distribution of the length of ballooning lines (*N* = 22). The corresponding underlying data can be found in S2 Data.

The separation of glands for a drag line and ballooning lines was observed in ballooning behaviors in the laboratory. The anchored drag line was connected to the anterior spinnerets, and ballooning fibers were spun from either 1 or both posterior and/or median spinnerets (see S5A and S5B Fig). The lengths of the ballooning fibers were measured, 3.22 ± 1.31 m (*N* = 22), from 22 spinning events of 2 crab spiders (16.4 and 18 mg) (see Fig 8). The maximum length of the spun ballooning lines was 6.2 m.

**Fig 9:**
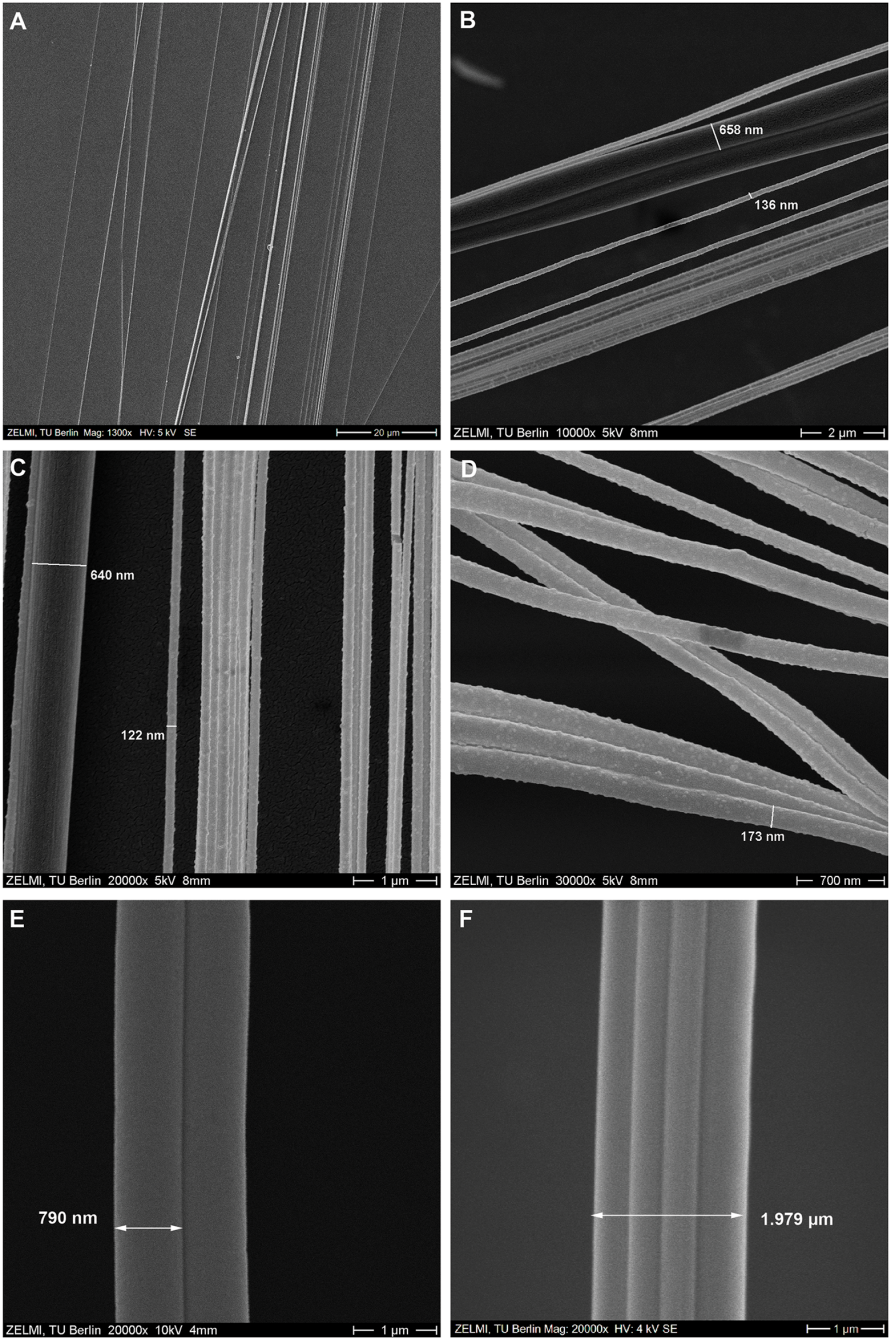
Scanning electron microscopic images of ballooning lines and drag lines. (A) Ballooning fibers of *X*. *cristatus* (1,300×). (B) Ballooning fibers of *X*. *audax* (10,000×). (C) Middle part of ballooning fibers of *X*. *audax* (20,000×). (D) Ballooning fibers of *X*. *cristatus* (30,000×). (E) One pair of drag fibers of *X*. *cristatus* (a weight of 18 mg) (20,000×). (F) Two pairs of drag fibers of *Xysticus* spp. (a weight of 15.6 mg), which attached together (20,000×).

The successfully collected ballooning fibers of both *X*. *cristatus* and *Xysticus* spp. were observed with the FESEM. Ballooning fibers consisted of 2 thick nanoscale fibers that were attached together (see Fig 9B and 9C) and many thin nanoscale fibers (see Fig 9A–9D). The 2 adult spiders, *Xysticus* species, spun 48–58 thin nanofibers and 2 thick nanofibers. The thickness of the thin nanofibers ranged from 121 to 323 nm, with an average of 211.7 ± 45.2 nm (*N* = 40). The thickness of the thick nanofibers was 698–768 nm, with an average of 722.2 ± 32.5 nm (*N* = 4) (see Table 2). The spun fibers were split independently (see Fig 9A–9D).

**Table 2:** Identification of the number and thickness of ballooning fibers through FESEM.

	Species	Weight	Thin nanoscale fibers		Thick nanoscale fibers	
			Number of fibers	Thickness of fibers	Number of fibers	Thickness of fibers
**Spider 1**	*Xysticus* spp *(f*.*)*	20.8 mg	58	192.3 ± 36.3 nm (*N* = 20)	2	734.0 ± 48.1 nm (*N* = 2)
**Spider 2**	*X*. *cristatus (f*.*)*	18 mg	48	231.1 ± 45.6 nm (*N* = 20)	2	710.5 ± 17.7 nm (*N* = 2)
**Average**	-	-	53 ± 7.1 (*N* = 2)	211.7 ± 45.2 nm (*N* = 40)	2 (*N* = 2)	722.2 ± 32.5 nm (*N* = 4)

Abbreviation: FESEM, field emission scanning electron microscope

On the other hand, drag lines consist of 1 pair of fibers (see Fig 9E) (sometimes 2 pairs, from left and right, see Fig 9F), which were spun from the major ampullate glands on the anterior spinnerets. The thickness of these major ampullate silks was about 500 to 800 nm (see Fig 9E and 9F).

### Aerodynamic environment on the short-grass field

**Fig 10:**
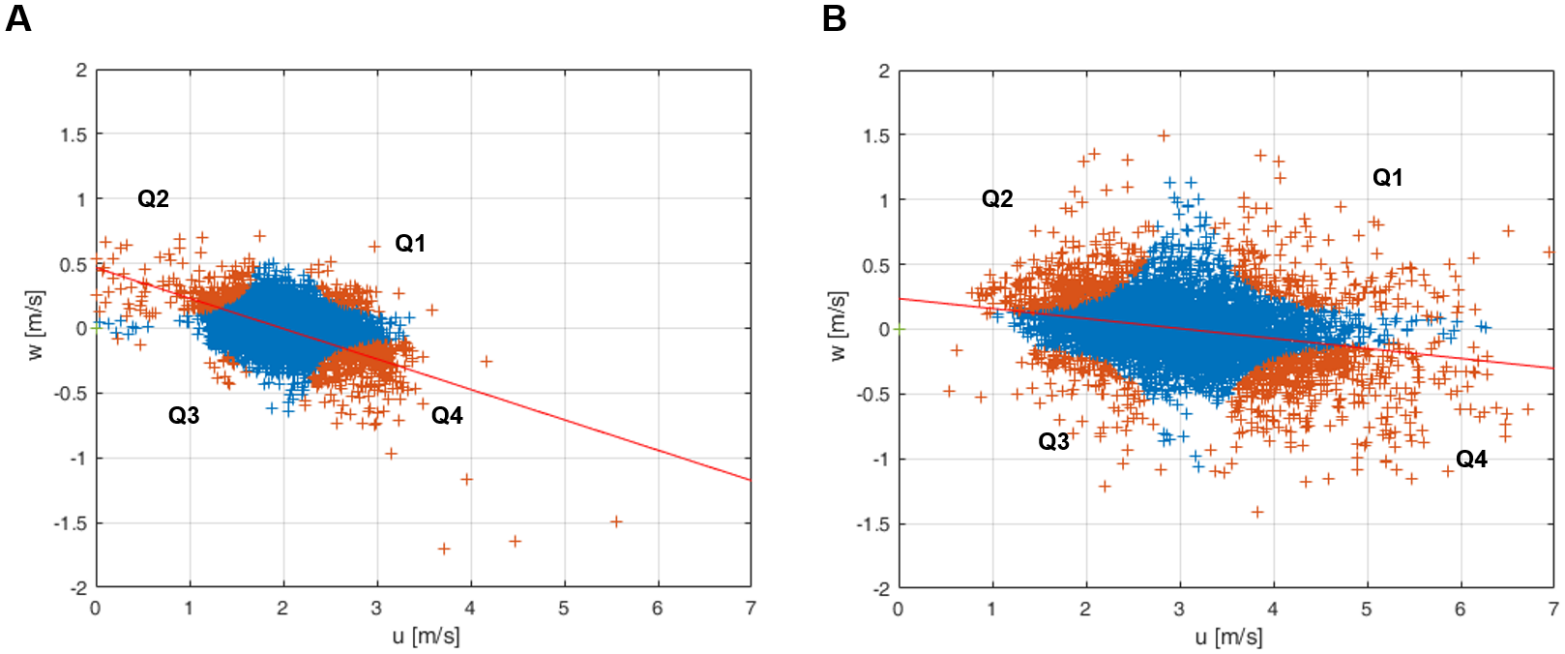
Horizontal and vertical components of the wind speeds for 5 min on u–w domain. (20-Hz sensing rate). (A) The case of 1.99 m s^−1^ mean wind speed (30 October 2016 12:39–12:45 LT). (B) The case of 3.07 m s^−1^ mean wind speed (29 October 2016 10:54–11:00 LT). Orange cross points: the quadrant data (Q1–Q4) of the measured wind speeds according to u′ and w′. Blue cross points: the ignored data regarding as a small-scale fluctuation (H < 1). Red lines are linear regression fit lines. The corresponding underlying data can be found in S3 Data.

Two data sets at the different mean wind speeds (1.99 m s^−1^ and 3.07 m s^−1^) for 5 min were collected. Usable updrafts were investigated in the turbulent atmospheric boundary layer. Each of the cases showed the vertical deviation of ±0.225 m s^−1^ and ±0.267 m s^−1^, respectively, which ranged from −0.5 to 0.5 m s^−1^ and from −0.6 to 0.7 m s^−1^ (see Fig 10A and 10B). The turbulent intensities were 21% and 23.7% in the mean wind speeds of 1.99 m s^−1^ and 3.07 m s^−1^, respectively. The vertical wind speed of both cases is negatively correlated to the horizontal wind speed. The slopes of regression fits are −0.235 (r = −0.575r) and −0.077 (r = −0.259), respectively. The horizontal wind speeds of both Q2s are positioned below the wind speed of 3 m s^−1^.

## Discussion

From our observation, the physical properties of ballooning silks were identified together with previously undescribed behaviors during ballooning: (i) an active sensing motion of the wind, (ii) an anchoring behavior during tiptoe takeoff, (iii) a tidying-up motion of an anchor line (drag line, safety line), and (iv) an outward stretching pose during a flight. These findings provide some clues and answers that were previously unsolved in spiders’ ballooning flight. The measured wind data also provide the possible mechanism from the viewpoint of the environment. We interpret as follows.

### Spider’s active sensing motion of the wind condition

**Fig 11:**
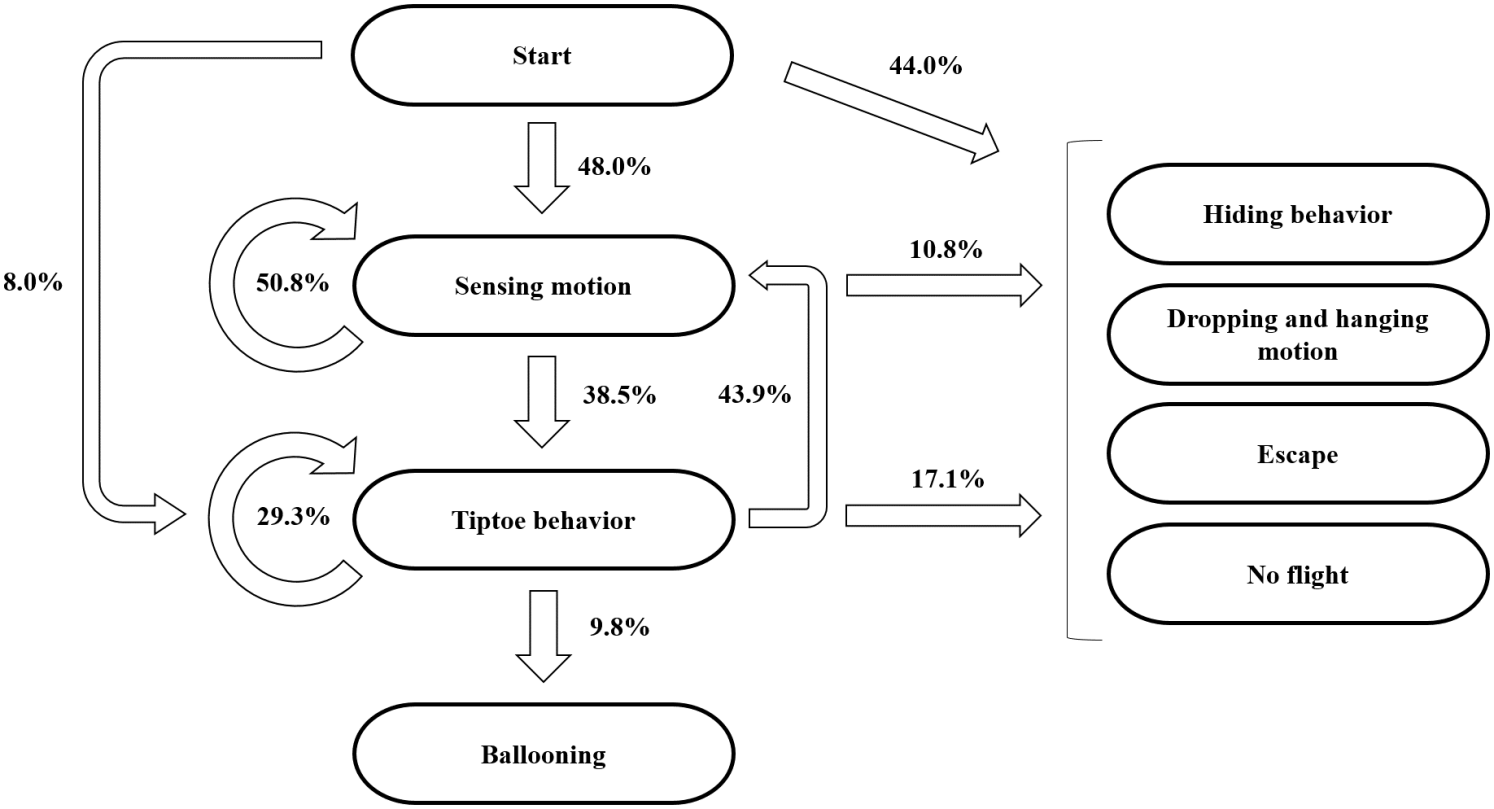
Takeoff process for tiptoe ballooning. The probabilities are calculated based on the total number of behaviors at each stage (see Fig 5B).

Crab spiders showed a motion that seemed like actively evaluating the meteorological condition before their takeoff. Normally, ballooning behavior is first triggered either by a warm ambient temperature or by a rapid increase in ambient temperature [31,36]. Additionally, if they are exposed to a wind that is slower than 3 m s^−1^ (favorable in 0.35–1.7 m s^−1^ wind speed for spiderlings), they show tiptoe behavior [1,3,36,37]. Until now, it had been thought that spiders sense the wind speed passively through the sensory hair (trichobothria) on their legs [17,31,45]. However, the present observations show that spiders may sense the aerodynamic condition of wind not just passively but rather actively, raising leg I high and shaking it. Strong interactive behavior transition between sensing motion and tiptoe motion (see Fig 5A) supports that active sensing motions are related to the spiders’ ballooning flight, because the tiptoe behavior is prerequisite behavior for ballooning takeoff (see Fig 11). The leg-raising behavior can be interpreted as follows: the spider enhances the sensibility of its sensory hairs by raising its legs upward in the outer region of the boundary layer, where airflows are faster than near the substrate and undisturbed by spiders’ bodies themselves. The first instars of the coccid *Pulvinariella mesembryanthemi* show a similar behavior by standing on their hind legs to capitalize on higher air speed in the thin boundary layer for their aerial dispersal [46].

The spider’s active sensing motion of the wind tells us two important things. First, in spiders’ ballooning, aerodynamic force maybe be a dominant factor. One hypothesis claims that an electrostatic charge on ballooning silks could generate lifting forces in the earth’s vertical atmospheric electrostatic field [22]. However, the leg-raising behavior indicates that, from the spider’s viewpoint, airflow is an important factor for its ballooning. Second, the spiders’ ballooning may not be random flight that simply relies on the random condition of the wind, but they may sense and evaluate the condition of the wind and wait for the appropriate moment to initiate ballooning. Thirty-eight percent of tiptoe behaviors lasted 10–65 s. This can be interpreted that there may be favorable wind conditions for spiders to balloon. If spiders evaluate wind conditions and wait for their aerial dispersal, this can be a distinct feature of spiders’ ballooning in contrast to other passive aerial dispersal, like that of seeds or aeroplanktons. Additionally, this evaluating behavior can also save their silk dopes during their takeoff trials by reducing failure cases, for example, by avoiding unfavorable wind conditions after spinning the ballooning silks.

In the near-surface atmospheric boundary layer, the updraft is not initiated by thermal buoyancy forces but is initiated by the shear winds, which generate shear-driven turbulent flows [47,48]. The generated turbulent eddies build intermittent updraft and downdraft regions, which drift to the downwind direction. If spiders sense either this moving updraft zone directly or the appropriate wind condition that can build this moving updraft structure, indirectly, the spiders’ sensing motion and timing of the decision may be helpful for their successful takeoff.

There are still other questions that can be asked. If spiders sense these wind conditions, what type of information do spiders need for their decision to balloon? From previous studies and the author’s observations, we deduce several factors. (i) Wind speed: A spider does not show tiptoe behavior under conditions of high wind speed, over 3 m s^−1^ [1,3,36,37]. (ii) A vertical wind speed: Favorable condition for ballooning, usually vertical acceleration of wind, persisting only for a few seconds. Under such a condition, the spider spins its silk fibers in wind rapidly and releases its substrate [20,36]. (iii) Wind direction: From our observation, spiders rotate their body in the direction of the wind as soon as they have evaluated the wind condition. Therefore, at least, spiders perceive wind direction. (iv) Wind fluctuation: For drop and swing dispersal, spiders were particularly more active with turbulent flows [49]. This shows that spiders can perceive the fluctuation of a turbulent flow. Suter showed that the ballooning site is usually laid within chaotic air flows [20]. Reynolds showed that the chaotic motion of turbulent flow reduces the terminal speed of ballooners and that this feature enables long permanence in the air [16]. Therefore, it can be deduced that spiders may sense the fluctuation of wind.

### Ballooning lines and anchored line

For crab spiders, ballooning lines were not identical with a drag line. The source of ballooning lines is not well known [3,19]. There is a report that some of primitive spiders—*Sphodros* spiderlings and *Ummidia* spiderlings, which do not use the complex preballooning behaviors such as tiptoe and rafting—use a drag line as a ballooning line, which is known as “suspended ballooning” [10]. Spiders drop down from the end of a branch, relying on their drag line. If there is a breeze, the drag line near its point of attachment to the platform would be sheared and drift through the air [28,50]. From this context, many previous studies regarded that spiders use their drag line for ballooning dispersal [16,17,22,23,38]. Some experiments substituted a drag line for a ballooning line [18,19,21]. However, Suter guessed from Tolbert’s observation that ballooning lines might be different from the drag line [3,18,20]. Our observation assures that in crab spiders, these ballooning lines are not identical with a drag line because those were spun from either or both of the median and/or posterior spinnerets (see S7A and S7B Fig). Normally, the drag line is spun from major ampullate glands in the anterior spinneret [51]. The crab spiders spun 48 to 58 thin nanofibers and 2 thick nanofibers. These thin nanoscale fibers seemed to be aciniform fibers (wrapping silk) from the median/posterior spinnerets. The thick nanoscale fibers seemed to be minor ampullate silks from the median spinnerets. Moon and An observed that one of the median spinnerets (left and right) of *Misumenops tricuspidatus* (crab spider) contains 2 pairs of ampullate glands and 20 (±3) pairs of aciniform glands, and one of its posterior spinnerets includes 50 (±5) pairs of aciniform glands [52]. Our observation in *Xysticus* spp. showed similar features to Moon and An’s observation (see S7C and S7D Fig).

The anchored drag lines are possibly normal drag lines (major ampullate silks), which a spider spins constantly while it is crawling and attaching its silk fibers to the ground surface for safety purposes. The anchored line was connected to the anterior spinnerets (see S7A Fig) and consisted of 2 fibers. From Osaki’s observation, drag lines are also composed of 2 fibers [53]. The anchored line can be observed in every ballooning behavior, in not only rafting takeoff [10] but also tiptoe takeoff.

Before the spinning motion of ballooning lines, there was a motion of a rear leg (leg IV) (see S5A and S5B Fig), resembling “wrap spinning,” during which spiders use leg IV to initiate ballooning lines by wiping their spinnerets [54]. However, in our case, it was obvious that the spiders did use their leg IV not to initiate ballooning lines but to tidy up the unfamiliarly positioned anchored line (safety line), which might have obstructed the spinning of ballooning silks (see S2A and S2B Fig and S2 Video). During the tiptoe takeoff, this anchored line is normally cut in a short time mechanically, when a spider drifts fast either horizontally or at a small inclination. But when a spider becomes slowly airborne with a very steep inclination because of a local updraft, this line bears until it is lengthened to 3–5 m. In our opinion, it is not stretched, but a spider lengthened the safety silk by spinning an additional safety line. The reason is that the maximum elongation of spider silks is limited by about 30% [55,56]. This means that spiders control their anchored line during the steep or slow takeoff.

### Nanoscale multifibers and ballooning flight

**Fig 12:**
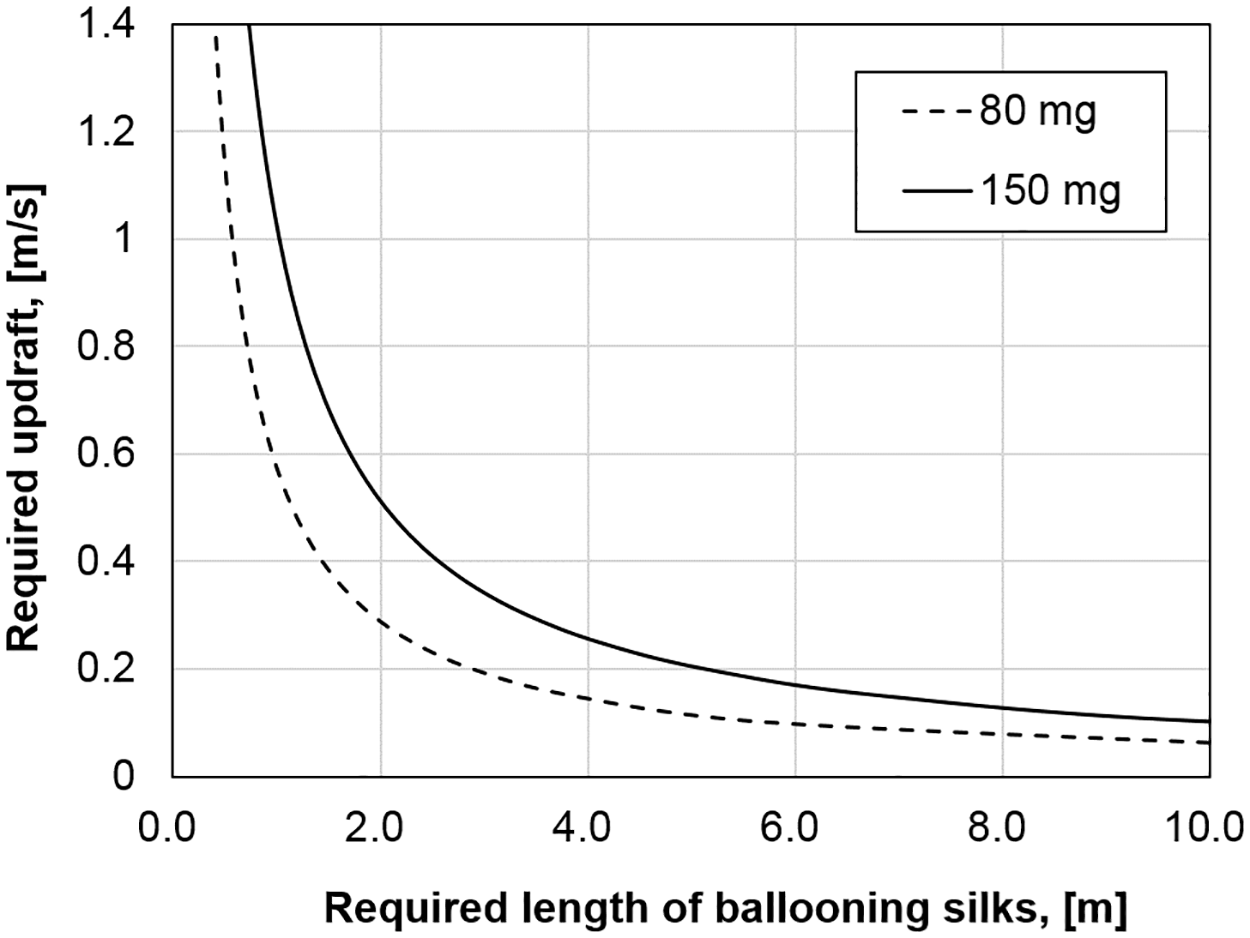
Required updraft wind speed and length of ballooning silks for the ballooning of 80–150 mg *Stegodyphus* spp. It is assumed that *Stegodyphus* spp. use 2 minor ampullate silks (2.1–2.9 μm thickness) and 78 aciniform silks (650–900 nm thickness) for their ballooning. The corresponding underlying data can be found in S4 Data.

While the ballooning of small spiders, which are lighter than 2.0 mg, was investigated by calculation [17], experiments [18,21], and observation [1,3], the plausibility of a large spider’s ballooning was not yet explained [17,39]. The mysterious flying behavior of large spiders can be explained by their nanoscale multifibers. From our wind tunnel test, we found that the *Xysticus* genus uses tens of nanofibers (diameters of 121 to 323 nm) for their aerial dispersal (see Fig 9A–9D). The number of ballooning fibers and their lengths were identified. Based on these measured values, the required updraft speed for the ballooning takeoff was calculated using modified Humphrey’s and Suter’s equations (Eqs [4–8]) [17,18]. The fluid-dynamic interaction between each fiber is not considered. The split case of fibers is calculated. For a crab spider weighing 10–25 mg, the required vertical wind velocities are, according to Humphrey’s theoretical formula, 0.08–0.20 m s^−1^ and, according to Suter’s empirical formula, 0.04–0.09 m s^−1^. These values are much smaller than the values 9.2–21.6 m s^−1^, which were calculated for *Stegodyphus* spp. [25,39]. We calculated the required length of ballooning fibers for *Stegodyphus* spp. according to the updraft air speeds (see Fig 12). Our result shows that *Stegodyphus* spp. can also balloon with relatively light updraft, 0.2–0.35 m s^−1^, with 3 m length and a total numbering 80 ballooning fibers. These results support Schneider’s observation that adult females of the *Stegodyphus* genus, 80–150 mg, balloon with at least tens to hundreds of threads [26]. In this calculation, the number of ballooning fibers for *Stegodyphus* genus is assumed conservatively that only 1 side (left or right) of the median and posterior spinnerets is used for spinning ballooning fibers. This number of fibers is estimated by counting the fiber glands of *S*. *mimosarum*’s spinnerets (the number of minor ampullate fibers is 2; the number of aciniform fibers is 78) [57]. This number is reasonable because *Xysticus* spp. spun a similar order of the number of fibers (2 thick fibers and 58 thin fibers, from our experiments). The thicknesses of a minor ampullate fiber and an aciniform fiber are assumed 80% and 25% of that of the major ampullate fiber, respectively. The thickness of a major ampullate fiber is estimated from Suter’s body weight and silk thickness relationship (Eq [8], [18]).

$$D_{H}=\sum_{i=1}^{n}\frac{2\pi ∙\mu ∙U∙l_{i}}{ln({2l}_{i}/d_{i})-0.72}\times 10^{6}$$ (4)

$$D_{S}=\sum_{i=1}^{n}11.5{∙\;l}_{i}∙\;U\;∙W_{eq,\;i}^{0.094}$$ (5)

$$U_{H}=\frac{W}{\sum_{i=1}^{n}\frac{2\pi \;∙\;\mu \;∙\;l_{i}}{ln({2l}_{i}/d_{i})\;-\;0.72}\times 10^{6}+\;1.94\;∙W^{0.366}}$$ (6)

$$U_{S}=\frac{W}{\sum_{i=1}^{n}11.5{∙\;l}_{i}∙W_{eq,\;i}^{0.094}+\;1.94\;∙W^{0.366}}$$ (7)

$$W_{eq,\;i}=\frac{\pi {\left(d_{i}\times 10^{6}\right)}^{2}+\;0.184}{0.0277}$$ (8)

*D*_*H*_: Drag of multiple fibers by Humphrey’s equation in μ*N*.

*D*_*S*_: Drag of multiple fibers by Suter’s equation in μ*N*.

*U*_*H*_: Required vertical wind speed for ballooning using Humphrey’s equation in m s^−1^. (The spider’s body drag is used from the Suter’s empirical relationship.)

*U*_*S*_: Required vertical wind speed for ballooning using Suter’s equation in m s^−1^.

*μ*: Dynamic viscosity of the air at 20°*C* (1.837 × 10^−5^ kg m^−1^ s^−1^).

U: Velocity of the air in m s^−1^.

*l*_*i*_: Length of i-th silk fiber in m.

*d*_*i*_: Diameter of i-th silk fiber in m.

*W*: Weight of the spider body in μ*N*.

*W*_*eq*,*i*_: Equivalent weight of the spider body corresponding to the diameter of a ballooning fiber in μ*N*.

*n*: Total number of ballooning fibers.

### Low Reynolds number fluid dynamics in a spider’s ballooning flight

The ballooning silks, exposed to the air, experience a low Reynolds number flow (Stokes flow, their Reynolds number is lower than 1). The upper limit of the Reynolds number is about 0.04 (*Re* = *ρVd* / *μ*; the air density, *ρ*: 1.225 kg m^−3^; the maximum possible velocity, *V*: 3 m s^−1^; the thickness of spider silk, *d*: 211 nm; the dynamic viscosity of air, *μ*: 1.837 × 10^−5^ kg m^−1^ s^−1^). The maximum possible speed that the thread experiences is assumed to be 3 m s^−1^ because a spider seldom flies above this wind speed. Once a spider is airborne, the relative air speed with respect to the silk is reduced. Therefore, the Reynolds number of spiders’ silks during their flight is much smaller than 0.04, and the spider’s flight is dominated by low Reynolds number fluid dynamics [58]. However, a large spider’s body shows much larger scales than those of a spider silk, not only in size but also in weight. In a free-fall case of a spider body without any silks, the Reynolds number is about 2,300 (*Re* = *ρV*_*t*_*D* / *μ*; $V_{t}=\sqrt{2\;mg\;/\;(\rho AC_{D})}$; a spider’s body is assumed as a 5 mm diameter, *D*, sphere with 25 mg mass, *m*; the projected area of a sphere, *A*: 19.6 mm^2^; the coefficient of a sphere, *C*_*D*_: 0.43; the terminal speed, *V*_*t*_: 6.2 m s^−1^) [59]. In this Reynolds number flow regime, the drag of a body is proportional to almost the square of relative wind speed, because the Reynolds number region of 5 < *Re* < 3,000 is categorized as moderate Reynolds numbers, in which inertial force of flow is still dominant in comparison with a viscous flow [60]. On the other hand, the drag of spider silk is proportional to relative wind speed because a viscous force is dominant in this low Reynolds number regime (*Re* ≪ 5) [60]. Therefore, the macroscale spider’s body, which means that a spider falls with acceleration without ballooning silks, is suspended by their high-tensile-strength nanoscale fibers that experience microscale fluid dynamics (low Reynolds number flow). This enables a spider to float in the air like a particle that experiences relatively low Reynolds number flow. The Reynolds number of a spider’s body with ballooning fibers is about 30 (*Re* = *ρVD* / *μ*; minimum possible velocity, *V*: 0.09 m s^−1^ by the lowest terminal speed from modified Suter’s formula). The utilization of low Reynolds number fluid dynamics in ballooning flight is different from flight mechanics of other membranous-winged insects’ flight, which mostly uses moderate or high Reynolds number aerodynamics, 10^3^ < *Re* < 10^5^ [61–63]. The low Reynolds number flight, which use a hairy structure, can also be found in that of thrips and parasitoid wasps [64–66]. While they produce drag during passive flight mode (parachuting) by using 200–300-μm-long stiff stae, spiders use about 3 m long flexible multifibers for ballooning flight. This scale difference (10^4^) enables large spiders’ ballooning (the weight of *Xysticus* spp. is 25 mg; the weight of *Encarsia formosa* [Chalcid wasp] is 2.55 μg) [64].

### Low wind speed and turbulence

**Fig 13:**
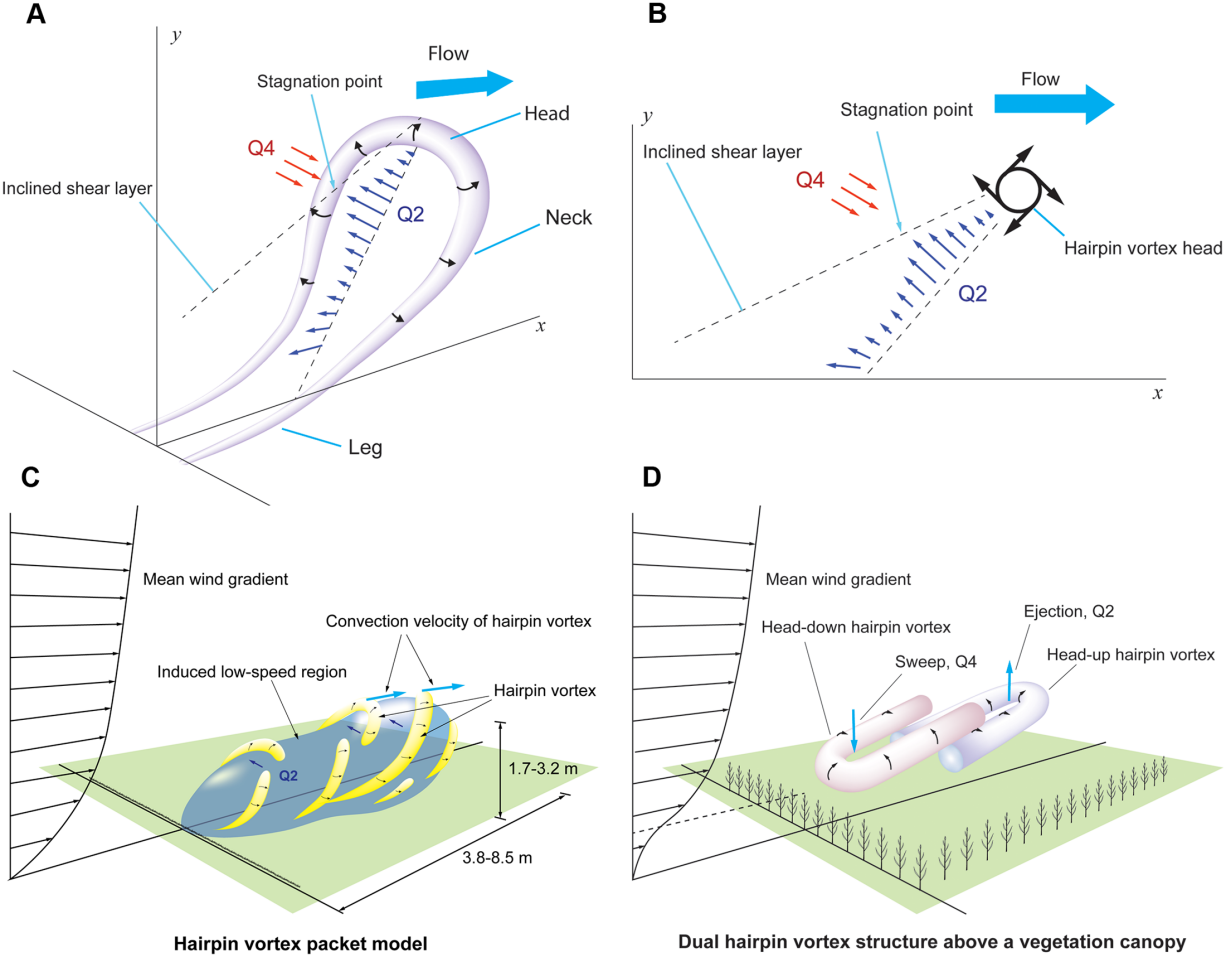
Schematic diagram of updraft generation by a vortex and vortices in near-surface atmospheric boundary layer. (A) Schematic diagram of a single hairpin vortex in the wall boundary layer. Q2 is an “ejection” region whose velocity vectors are u′ < 0 and v′ > 0. Q4 is a “sweep” region whose velocity vectors are u′ > 0 and v′ < 0. (B) Cross-section of the x-y plane of the hairpin vortex. (C) Schematic diagram of the hairpin vortex packet. Yellow colors mean hairpins or cane-type vortices. Blue region means low momentum region, which contains upward air currents. (D) Coherent structure, “dual hairpin vortex,” on the plant field. Head-down hairpin vortex produces “sweep” event. Head-up hairpin vortex produces “ejection” event. (A, B) Redrawn from [68]. (C) Redrawn from [68, 69]. (D) Redrawn from [70].

The measured vertical wind speeds of updrafts ranged 0–0.5 m s^−1^ at the mean wind speed of 1.99 m s^−1^ and 0–0.7 m s^−1^ at the mean wind speed of 3.07 m s^−1^. These values are enough for spiders’ ballooning, the required vertical wind speeds of which were 0.04–0.2 m s^−1^ for 10–25 mg *Xysticus* spp. and 0.2–0.35 m s^−1^ for 80–150 mg *Stegodyphus* spp. The observed negative correlation between vertical wind speed and horizontal wind speed in the turbulent boundary layer, which has been also studied in the fields of fluid dynamics and meteorology [42–44], suggests to us that the reason spiders show their preballooning behavior at a low-wind-speed regime (smaller than 3 m s^−1^) is because they may use the “ejection” regimes in turbulent flow, which contain updraft components and are induced by a “coherent structure” near the surface boundary layer. A shear wind on a planted field, a meadow, or relatively short plants form an organized structure called the “coherent structure” (possibly “hairpin vortex” or “horseshoe vortex,” see Fig 13A and 13B; “hairpin vortex packets” over relatively short plants and “dual hairpin vortex structures” over planted field [see Fig 13C and 13D]) [42,67–71]. These structures intermittently produce up- and downdrafts [42,68,70]. The interesting point is that these updrafts are highly correlated with a decrease in wind speed, which is categorized as Q2 (*u*′ < 0 and *v*′ > 0), an “ejection” region in a quadrant analysis [42–44] (see Fig 10). Therefore, the phenomenon of spiders usually ballooning in the low-wind-speed regime (lower than 3 m s^−1^) could be explained with this organized structure in the atmospheric turbulent flows above the ground. However, the frequency and duration of these updrafts are not well known.

### Shear flow and the spider’s posture

While Reynolds postulated the tangled shape of ballooning fibers in his simulation, the diagonally lying shape of the spider’s ballooning fibers was observed in our field observations in the Teltow Canal (see Fig 7) [16]. The difference between our observation and Reynolds’ simulation is caused by the fact that while Reynolds introduced homogeneous turbulence in his simulation, the real turbulence near the surface boundary layer includes instantaneous horizontal wind shears that are mostly induced by vertical differences in wind speed [71,72]. This diagonally stretched shape of ballooning fibers may be caused by the shear wind in the atmospheric boundary layer. We think that this may be helpful for long-endurance ballooning flight because horizontally stretched silks produce more drag, up to a factor of 2, than vertically distributed shapes of silk, because of an anisotropic drag of silks in a low Reynolds number flow [73] (see S8A–S8C Fig).

The observed fact, that spiders outstretch all legs during their flight, is puzzling because of its small drag ratio compared to the drag of a spider’s ballooning silks. Suter concluded that when a spider uses a relatively short length of silk, the influence of posture on its terminal speed is greater than when the silk is very long [19]. However, our observation shows that spiders spin abundant ballooning fibers. From Suter’s empirical formula, spiders’ (weighing 10 to 25 mg) body drags per unit velocity, which are 10.4–14.5 μN m^−1^ s, are just 0.4–0.56% of the whole drag (silks + body) per unit velocity, 2576.5–2580.5 μN m^−1^ s. Even if a spider stretches its legs outward, the percentage of body drag is still 2.0–2.8% (5-fold change is applied, from Suter’s research) [18,19]. Despite such a small drag effect of spiders’ posture, spiders stretch their legs outward during flight (see S6C and S6D Fig). Then the question arises, What role do the stretched legs play in ballooning flight? These should be studied as a future work.

## Conclusion and outlook

Studying ballooning mechanics in spiders can be helpful for understanding not only the ecological influence of spiders’ dispersal but also efficient passive transport of particles using air or oceanic currents.

By observing the ballooning behavior in relatively large spiders (10–25 mg *Xysticus* spp.) in the field and in the laboratory, we have revealed that the large spiders’ ballooning, even that of 80–150 mg *Stegodyphus* spp., is possible with the help of tens of multiple nanoscale fibers. The observed ballooning lines were not identical to the drag line, which has been regarded as a ballooning line. From the observation of spinning behaviors and morphological dimensions of fibers, we concluded that these ballooning silks are pairs of minor ampullate silks and multiple aciniform silks, which are usually used in other species as wrapping silks. *Xysticus* spp., however, used these silks for their aerial dispersal. Spiders also showed an interesting behavior (active sensing motion) such as evaluating the wind conditions before their ballooning behavior. This behavior may save spiders’ silk dopes, which can be consumed during their takeoff trials.

Two major features in the physics of ballooning are suggested from the study. First, atmospheric shear flow, which is a major part of the flow structure in the turbulent boundary layer, may be helpful for the high buoyant capability of a ballooning structure because horizontally/diagonally stretched silks produce more drag than vertically distributed shapes of silks. Second, spiders may use the updrafts that are induced by “coherent structures” in the turbulent atmospheric boundary layer. From the measured wind data, we showed that these updrafts are correlated with lower wind speeds. Therefore, this hypothesis is expected to explain why spiders usually balloon when the wind speed is lower than 3 m s^−1^. However, these suggestions should be studied further for theoretical firmness.

Whether or not vertical wind speed and fluctuation of wind influence on spiders’ evaluation processes for ballooning, how multiple fibers form a triangular sheet without entanglement during the takeoff phase (do any electrostatic forces act between fibers [11, 22, 26], or can the bundle of fibers be unfolded only with the help of wind fluctuation?), and why spiders stretch their legs outward during their flight are questions that still remain. These could be interesting topics for future research.

## Supporting information

S1 Fig Ballooning behavior of a male crab spider. The spider shows the tiptoe behavior and spins ballooning silks.
(TIF)

S2 Fig Sequential sketches of “tiptoe” takeoff in the field experiment. Red lines: an anchored line (a drag line). Blue lines: ballooning lines. (A) A spider, *Xysticus* spp., tries to hold the anchored line with 1 of legs IV. (B) The spider holds the anchored line and then puts it on the substrate. (C) The spider first spins a single or a few fibers. (D) And then, the spider spins many split fibers. (E) If the wind condition is appropriate, the spider releases the substrate. (F) In a very short time, the anchored line is cut. The crab spider becomes airborne.
(TIF)

S3 Fig Sequential sketches of “rafting” takeoff in the field experiment. Red lines: an anchored line (a drag line). Blue lines: ballooning lines. (A)(B) A spider, *Xysticus* spp., drops down about 0.4–1.1 m, relying on its anchored line (a drag line). (C) The spider first spins a single or a few fibers downstream of the wind. (D) And then, the spider spins many fibers continuously. (E)(F) The spun ballooning lines slowly curved upward. At some point, the anchored line near the spinnerets is cut, and the spider balloons.
(TIF)

S4 Fig The sketch describes the position of the hair dryer. The mixed air zone generates 28–33 °C warm air and fluctuating updraft (horizontal mean wind speed: 0.58 m s^−1^, vertical mean wind speed: 0.40 m s^−1^).
(TIF)

S5 Fig Tidying-up motion of safety line (anchored line) with leg IV. Two independent events; a front view (A), a side view (B). Both spiders hold their safety line and then put it on the substrate before spinning of ballooning lines.
(TIF)

S6 Fig Spiders’ posture in takeoff and flight. (A, B) An anchored line was found during a tiptoe takeoff. As soon as spiders were airborne, they stretched the legs outward. (C) To ensure the behavior of outstretched legs during flight, the pose of a spider was observed during its gliding phase. (D) The spider kept its legs outstretched.
(TIF)

S7 Fig Spinning behavior for ballooning and scanning electron microscopic images of spinnerets. (A) Spinning motion of ballooning silks in front of the open jet wind tunnel. Ballooning lines were spun from either or both of the median or/and posterior spinnerets. (B) Spinnerets of *X*. *cristatus* through FESEM. (C) Median spinneret of *Xysticus* spp. Two minor ampullate glands and 10 aciniform glands are distributed on the median spinneret. (D) A number of aciniform glands on the posterior spinneret. FESEM, field emission scanning electron microscope.
(TIF)

S8 Fig Schematic diagrams of the ballooning structure in a shear flow. (A) Ballooning structure in a shear flow. (B) Drift of a ballooning structure along with wind. The upper and lower parts of the silk are exposed to the flow fields, which exert to the other directions. (C) The ballooning structure exposed in a shear flow is stretched horizontally. ***D*_*S*_**, horizontal component of drag on the spider’s body; ***h***, height; ***V*_*D*_**, drift speed of a ballooning structure; ***V*_*wG*_**, wind speed profile relative to ground; ***V*_*wR*_**, wind speed profile relative to a ballooning structure.
(TIF)

S1 Video Sensing motion of a crab spider before tiptoe motion. (MP4)

S2 Video Tidying-up motion of an anchored line before spinning ballooning lines. (MP4)

S3 Video A preballooning behavior of a crab spider, *Xysticus* spp. (MP4)

S1 Data Statistical data of preballooning behaviors. (XLSX)

S2 Data Physical properties of ballooning fibers. (XLSX)

S3 Data Three-dimensional wind velocity data on a flat grass field. (XLSX)

S4 Data Required vertical airspeed for ballooning. (XLSX)
