# Supplementary figures and images for: An observational study of ballooning in large spiders: Nanoscale multifibers enable large spiders’ soaring flight
Source: PLoS Biol. 2018 Jun 14;16(6):e2004405. doi: 10.1371/journal.pbio.2004405 (PMC6001951; doi:10.1371/journal.pbio.2004405)

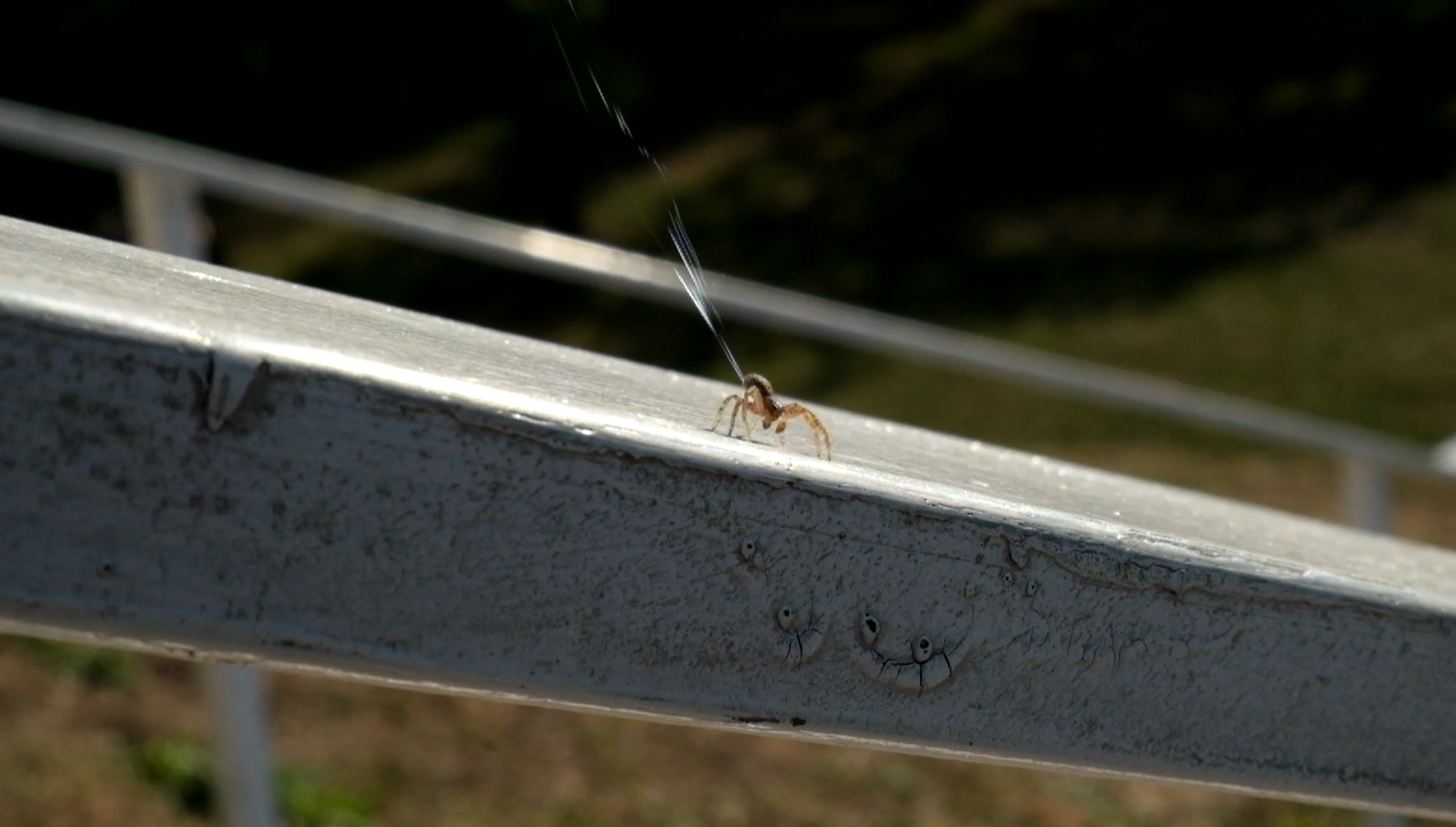

Supplement: S1 Fig — The spider shows the tiptoe behavior and spins ballooning silks. (TIF) [file pbio.2004405.s001.tif]

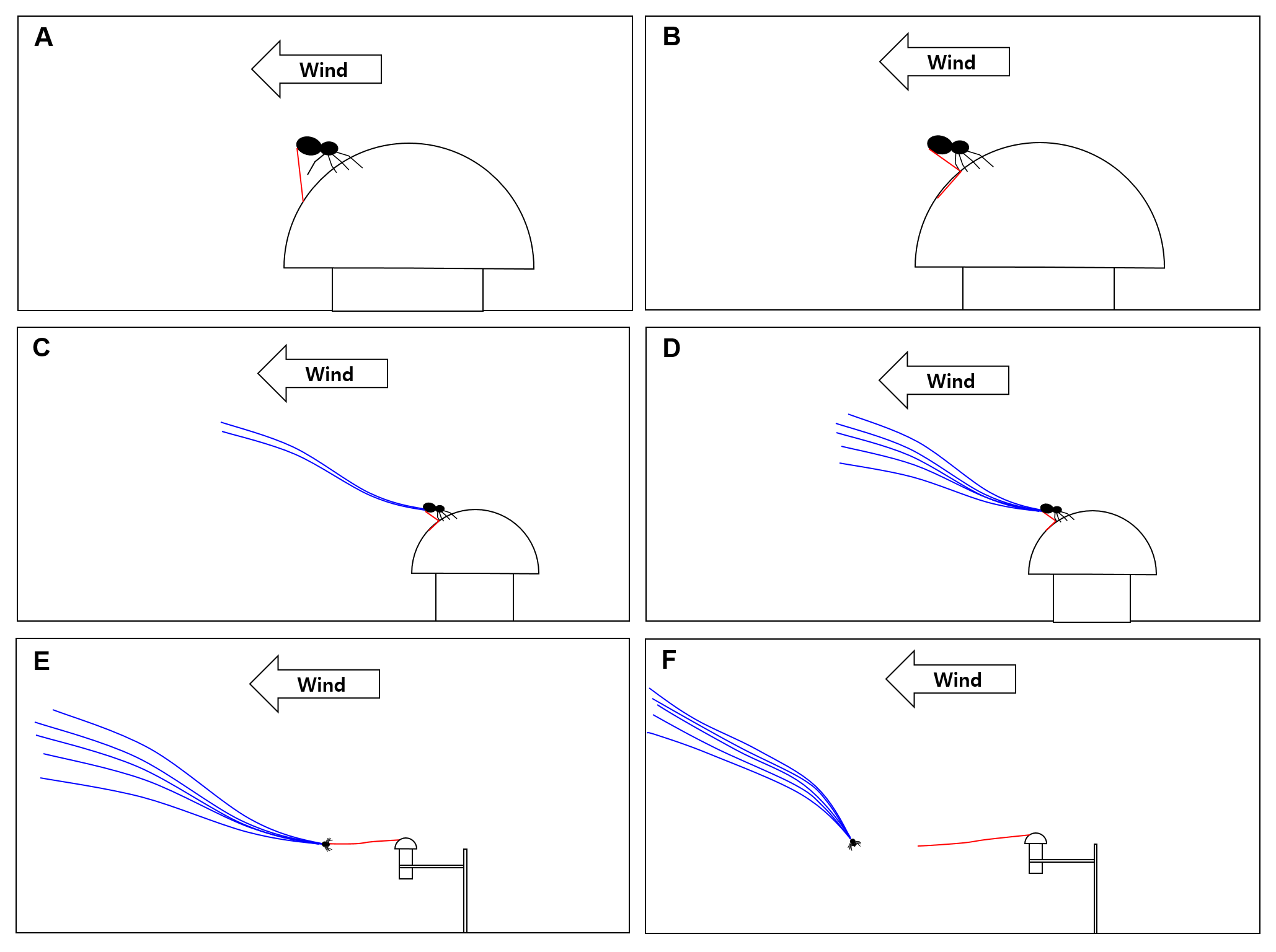

Supplement: S2 Fig — Red lines: an anchored line (a drag line). Blue lines: ballooning lines. (A) A spider, Xysticus spp., tries to hold the anchored line with 1 of legs IV. (B) The spider holds the anchored line and then puts it on the substrate. (C) The spider first spins a single or a few fibers. (D) And then, the spider spins many split fibers. (E) If the wind condition is appropriate, the spider releases the substrate. (F) In a very short time, the anchored line is cut. The crab spider becomes airborne. (TIF) [file pbio.2004405.s002.tif]

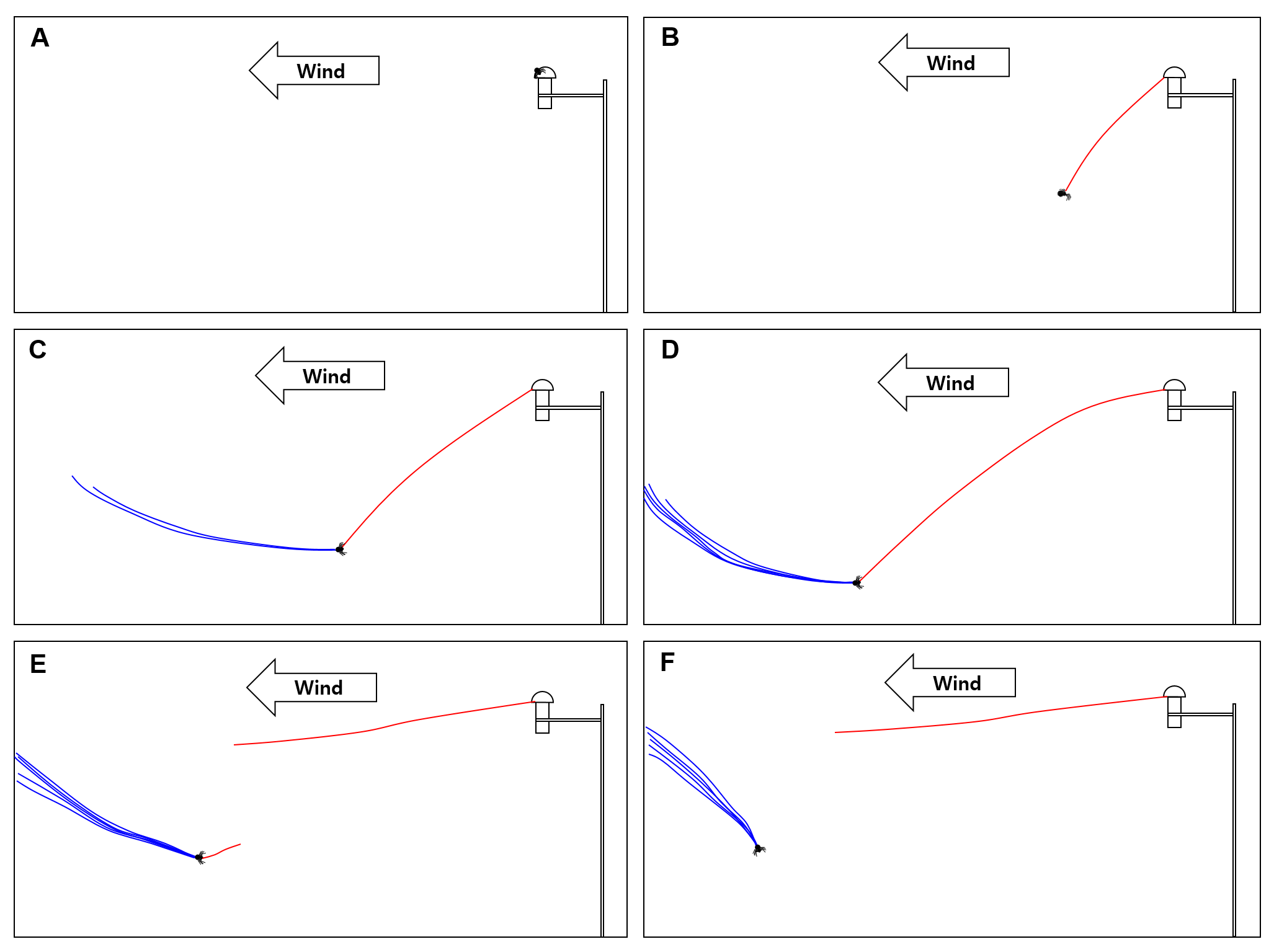

Supplement: S3 Fig — Red lines: an anchored line (a drag line). Blue lines: ballooning lines. (A)(B) A spider, Xysticus spp., drops down about 0.4–1.1 m, relying on its anchored line (a drag line). (C) The spider first spins a single or a few fibers downstream of the wind. (D) And then, the spider spins many fibers continuously. (E)(F) The spun ballooning lines slowly curved upward. At some point, the anchored line near the spinnerets is cut, and the spider balloons. (TIF) [file pbio.2004405.s003.tif]

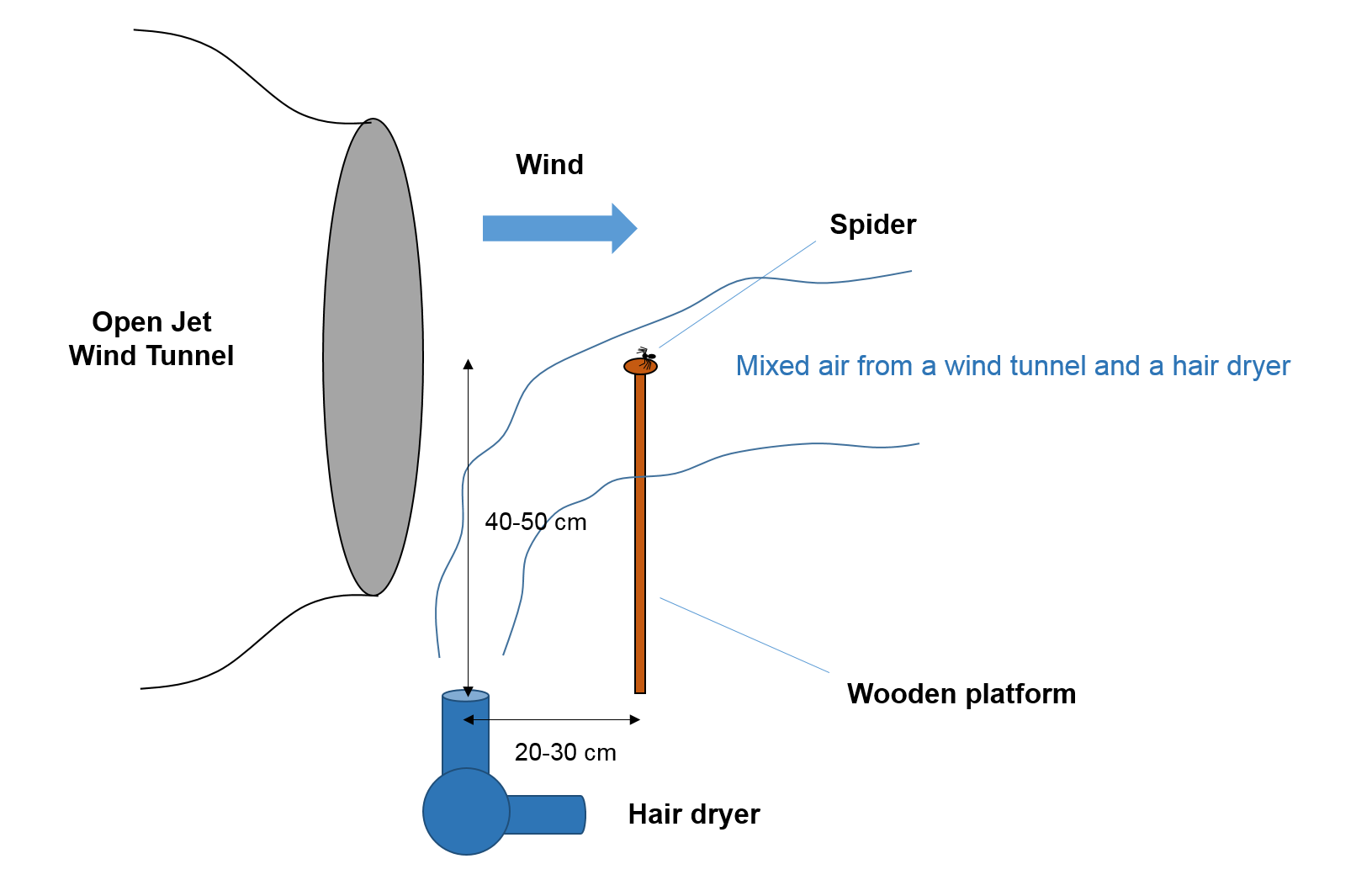

Supplement: S4 Fig — The mixed air zone generates 28–33 °C warm air and fluctuating updraft (horizontal mean wind speed: 0.58 m s−1, vertical mean wind speed: 0.40 m s−1). (TIF) [file pbio.2004405.s004.tif]

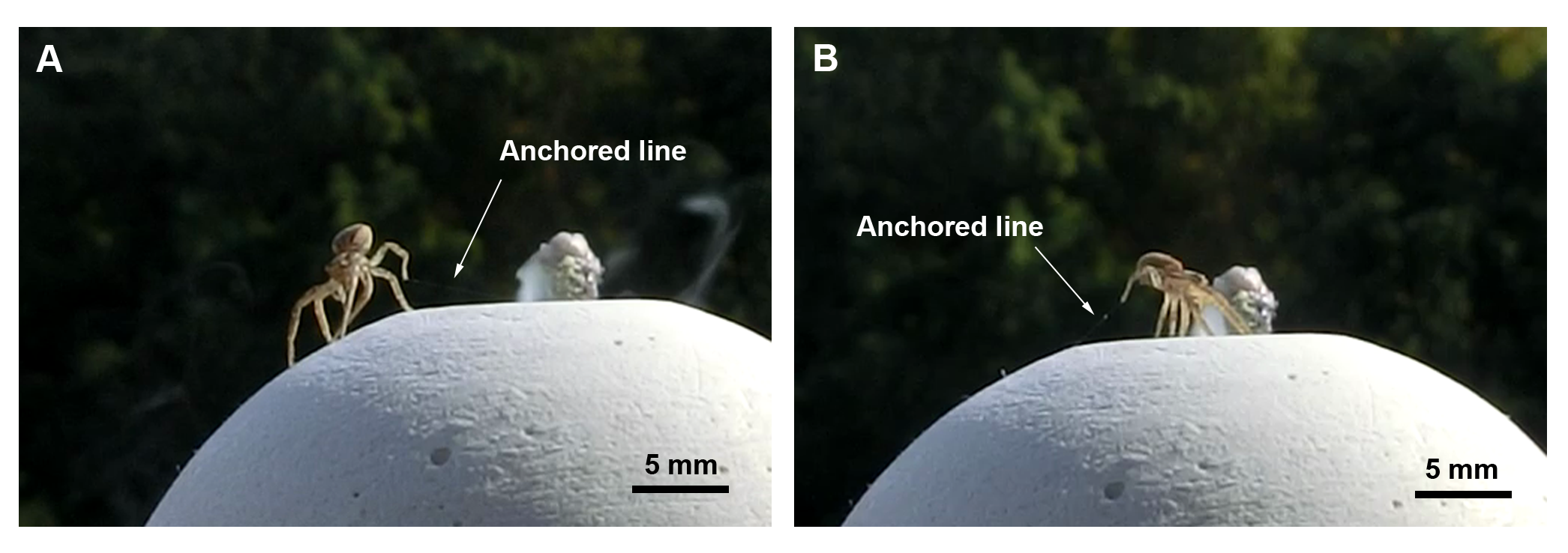

Supplement: S5 Fig — Two independent events; a front view (A), a side view (B). Both spiders hold their safety line and then put it on the substrate before spinning of ballooning lines. (TIF) [file pbio.2004405.s005.tif]

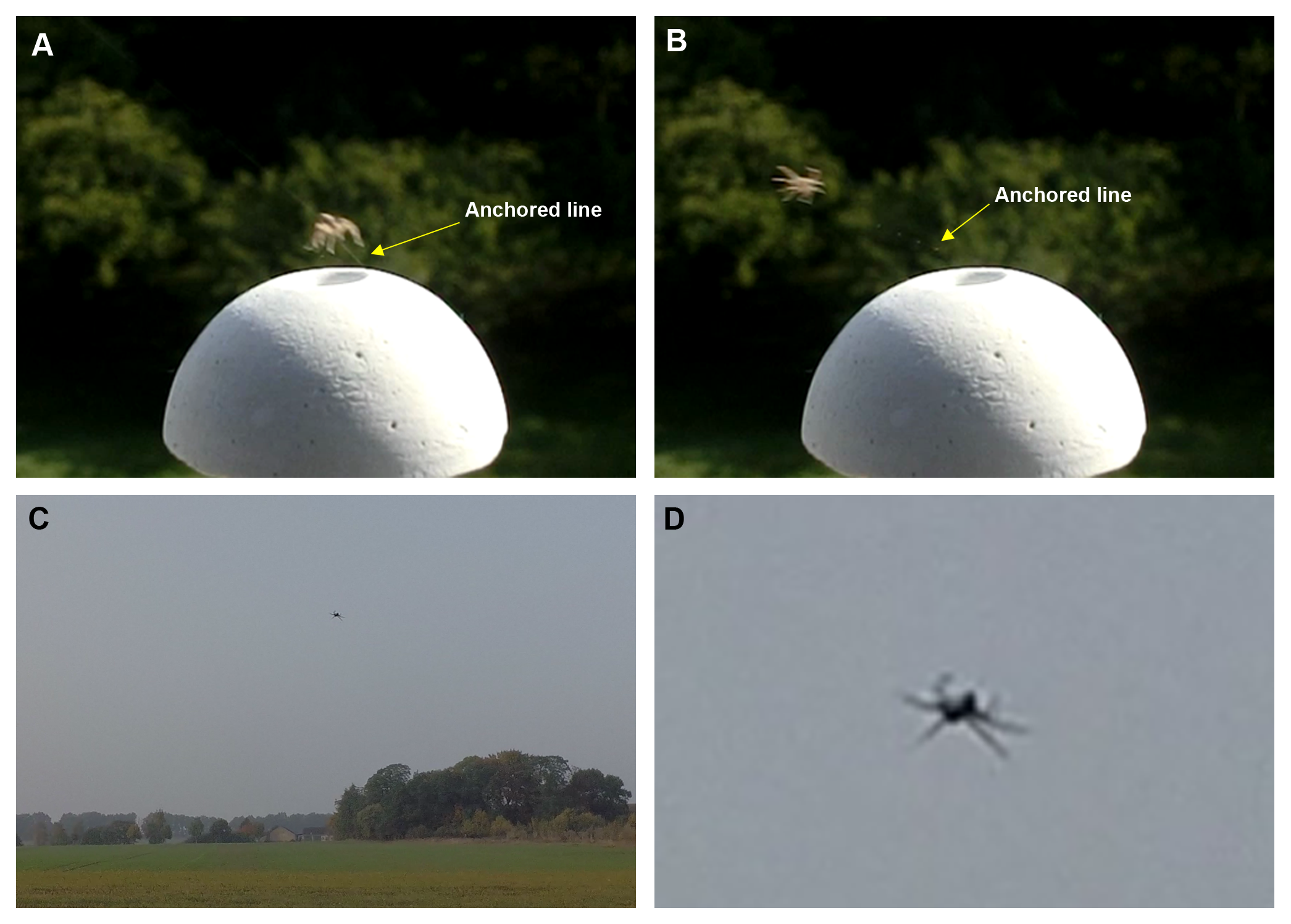

Supplement: S6 Fig — (A, B) An anchored line was found during a tiptoe takeoff. As soon as spiders were airborne, they stretched the legs outward. (C) To ensure the behavior of outstretched legs during flight, the pose of a spider was observed during its gliding phase. (D) The spider kept its legs outstretched. (TIF) [file pbio.2004405.s006.tif]

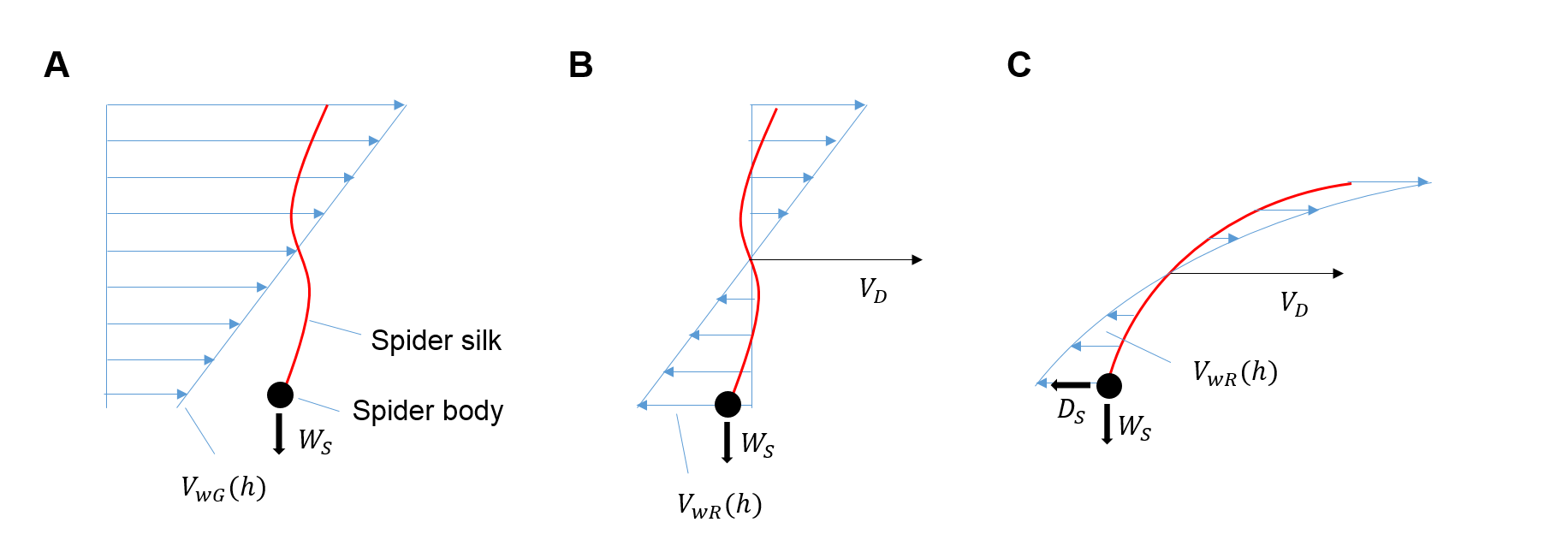

Supplement: S8 Fig — (A) Ballooning structure in a shear flow. (B) Drift of a ballooning structure along with wind. The upper and lower parts of the silk are exposed to the flow fields, which exert to the other directions. (C) The ballooning structure exposed in a shear flow is stretched horizontally. DS, horizontal component of drag on the spider’s body; h, height; VD, drift speed of a ballooning structure; VwG, wind speed profile relative to ground; VwR, wind speed profile relative to a ballooning structure. (TIF) [file pbio.2004405.s008.tif]
